# Bankable human iPSC-derived retinal progenitors represent a valuable source of multipotent cells

**DOI:** 10.1038/s42003-023-04956-2

**Published:** 2023-07-21

**Authors:** Sandy Gozlan, Vivien Batoumeni, Tara Fournier, Céline Nanteau, Anais Potey, Marilou Clémençon, Gaël Orieux, José-Alain Sahel, Olivier Goureau, Jérôme E. Roger, Sacha Reichman

**Affiliations:** 1 Sorbonne Université, INSERM, CNRS, Institut de la Vision, F-75012 Paris, France; 2 CHNO des Quinze-Vingts, INSERM-DGOS CIC 1423, F-75012 Paris, France; 3 Fondation Ophtalmologique Adolphe de Rothschild, F-75019 Paris, France; 4 Department of Ophthalmology, The University of Pittsburgh School of Medicine, Pittsburgh, PA 15213 US; 5 Paris-Saclay Institute of Neuroscience, CERTO-Retina France, CNRS, Université Paris-Saclay, 91400 Saclay, France

**Keywords:** Multipotent stem cells, Stem-cell differentiation

## Abstract

Retinal progenitor cells (RPCs) are the source of all retinal cell types during retinogenesis. Until now, the isolation and expansion of RPCs has been at the expense of their multipotency. Here, we report simple methods and media for the generation, expansion, and cryopreservation of human induced pluripotent stem-cell derived-RPCs (hiRPCs). Thawed and passed hiRPCs maintained biochemical and transcriptional RPC phenotypes and their ability to differentiate into all retinal cell types. Specific conditions allowed the generation of large cultures of photoreceptor precursors enriched up to 90% within a few weeks and without a purification step. Combined RNA-seq analysis between hiRPCs and retinal organoids identified genes involved in developmental or degenerative retinal diseases. Thus, hiRPC lines could provide a valuable source of retinal cells for cell-based therapies or drug discovery and could be an advanced cellular tool to better understand retinal dystrophies.

## Abstract (web summary)

An in vitro method can expand and bank retinal progenitor cells from hiPSC-derived retinal organoids, representing a potential source of retinal cells for future cell-based therapies or drug discovery models.

## Introduction

In mammals, neuroregenerative capacity is poor, including that of the human retina. Therefore, retinal dystrophies (RDs) that cause the definitive loss of neural cells typically result in permanent visual impairment. Preventing degeneration and rescuing the degenerated retina are major challenges for which stem cell-based therapies show promise^1,2^. Many methods have emerged to generate human induced pluripotent stem cell (hiPSC)-derived retinal cells and tissues, such as retinal organoids (ROs), for cell therapy, drug discovery^3,4^, or to better understand inherited RDs by modeling the progression of degeneration. However, the selection of specific cells within ROs requires sorting, limiting the scale-up of production. Part of the challenge of using hiPSC derivatives in cell-based therapies is the production of a large number of specific and identical cells. Because new therapies are cell-intensive, the simple and efficient production of such cells is required. To meet these needs, we proposed the use of mitotic and multipotent retinal progenitor cells (RPCs) isolated from early ROs. Indeed, RPCs are the source for the generation of all retinal cell types during retinogenesis^5^. Initially isolated from human fetal retinas^6^, RPCs can be generated from differentiation protocols using human iPSCs^7–11^. Although RPCs appear to be expandable, numerous studies have described the loss of their multipotency during culture^6^ or following multiple passages^12–14^. Here, we describe a method and an innovative RPC-dedicated medium (RPCM) that allow the amplification of hiPSC-derived RPCs (hiRPCs) in adherent culture condition while maintaining their multipotency. The RPCM-based culture contains five extrinsic factors (CHIR99021, PURMORPHAMINE, ATP, FGF2, and EGF) that act on pathways involved in retinal development and allowed several passages of hiRPCs for the production of millions of cells from cryopreserved stocks. RNAseq analysis confirmed preserved multipotency, with a preneurogenic phenotype, of hiRPCs expanded in RPCM. From cryopreserved stocks, hiRPCs were able to differentiate into all retinal cell types, such as retinal pigmented epithelium (RPE), retinal ganglion cells (RGCs), horizontal cells, amacrine cells and photoreceptor precursor cells (PPCs) within a few weeks as well as mature photoreceptors expressing rod and cone opsins, bipolar cells and Müller glial cells in long-term culture. Moreover, we describe defined culture conditions that allow the generation of enriched cultures of hiRPC-derived PPCs (hiPPCs) of up to 90% in 2 weeks without a purification step. Thus, we describe methods and media to isolate, expand, and cryopreserve bankable hiRPCs as a powerful cellular tool. This multipotent cell line, as well as cell derivatives, could be used for developmental research, cell-based therapy, and drug discovery in the foreseeable future.

## Results

### Generation of the human iPSC-derived retinal progenitor cell line

**Fig. 1:**
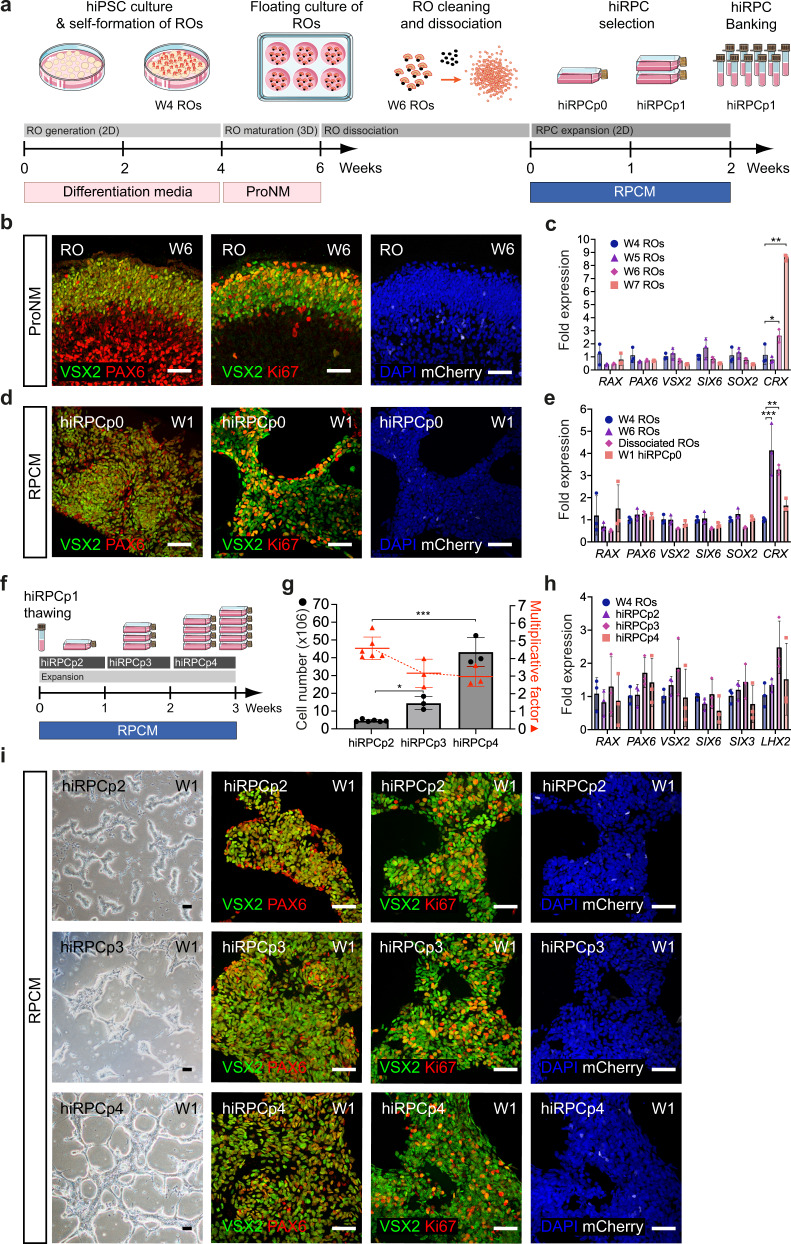
Generation and characterization of a retinal progenitor cell line from iPSC-derived retinal organoids. **a** Schematic diagram illustrating the protocol for the generation of RPCs from hiPSCs and cell banking. **b** Immunofluorescence staining of cryosectioned ROs at W6 for VSX2, PAX6, Ki67, and endogenous mCherry staining. **c** RT-qPCR analysis of eye-field transcription factors (EFTFs; *RAX, PAX6, VSX2, SIX6*), SOX2, and *CRX* in ROs between W4 and W7. Data are normalized to that of W4 ROs and presented as mean ± SD (n = 3 per time point). **d** Immunofluorescence staining of hiRPCp0 after one week of culture (W1) in RPCM for VSX2, PAX6, and Ki67 and endogenous mCherry staining. **e** RT-qPCR analysis of EFTFs (*RAX, PAX6, SOX2, SIX6*), *VSX2*, and *CRX* in ROs at W4 and W6, dissociated ROs at day 1, and hiRPCp0 expanded for one week (W1) in RPCM. Data are normalized to that of W4 ROs and presented as mean ± SD (n = 3 per time point). **f** Schematic diagram illustrating hiRPC expansion in RPCM from cryopreserved hRPCp1 to hiRPCp4 in three weeks. **g** Multiplication factor (red) and hiRPC number (gray histograms) after successive passages. Data are normalized to that of seeded hiRPCp2 at D0 and presented as mean ± SD (n = 6 for hiRPCp2 and n = 3 for hiRPCp3-4). **h** RT-qPCR analysis of EFTFs (*RAX, PAX6, SIX6, SIX3, LHX2)* and *VSX2* in W4 ROs and hiRPCp2 to hiRPCp4. Data are normalized to that of W4 ROs and presented as mean ± SD (n = 3 per time point). **i** hiRPC characterization after one week (W1) of culture in RPCM by phase-contrast and brightfield microscopy (left panels) and immunofluorescence staining of hiRPCp2, hiRPCp3, and hiRPCp4 for *VSX2, PAX6*, and *Ki67* and endogenous expression of mCherry. One-way ANOVA followed by a Dunnett’s multiple comparison test (**c**, **e**, **g**, **h**). Comparison to W4 ROs (**c**, **e**, **h**) or hiRPCp2 (**g**). ***p  <  0.001; **p  <  0.01; *p  <  0.05. Nuclei were counterstained with DAPI (blue). hiPSC-5FC-derived cells. Scale bar: **b**, **d**, **i**, 50 µm.

ROs were generated from the human fluorescent reporter AAVS1::CrxP_H2BmCherry-hiPSC-5FC line^15^ and from the nonfluorescent hiPSC-2^16^ and hiPSC-5F^17^ lines using our previously reported protocol^16,18^ (see materials and methods). Data presented in main figures were done with the fluorescent reporter hiPSC-5FC line to target hiPPCs through the mCherry expression. Thus, after 4 weeks (W4) of hiPSC differentiation, ROs were picked and cultured under floating-culture conditions until W6 (Fig. 1a). Human iPSC-derived RPCs were isolated from ROs at W6, which are composed of two major cell types organized in two layers (Fig. 1b). The internal layer contains PAX6^+^/VSX2^-^ cells, mainly corresponding to RGCs, the first post-mitotic differentiated cell type^7,16^ (Supplementary Fig. 1). The external layer is composed of a cell population co-expressing PAX6^+^/VSX2^+^ and the mitotic marker KI67, representing RPCs (Fig. 1b, Supplementary Fig. 2). RT-qPCR analysis confirmed that the progenitors present in ROs at W6 still expressed the eye-field transcription factors (EFTFs) *RX, PAX6, SIX6*, *SOX2* and the optic vesical specific transcription factor *VSX2* while retaining the expression of the photoreceptor marker CRX at a basal level relative to ROs at W7 (Fig. 1c). Moreover, the absence of endogenous mCherry staining, illustrating the absence of cells committed towards the photoreceptor lineage, confirmed the undifferentiated state of the RPC layer at W6 (Fig. 1b, Supplementary Fig. 2). Before RPC isolation, the pigmented portion of W6 ROs was removed to avoid any RPE contamination in the downstream hiRPC cultures (Fig. 1a). This cleaning step is not possible before W6 because of the small size of the ROs and the absence of the pigmentated portion. After papain-based dissociation of cleaned ROs, seeded cells were designated as hiRPCp0 and cultured in a new specific RPC-dedicated medium (RPCM) that acts on multipotency and proliferation by the addition of the five extrinsic factors CHIR99021, PURMORPHAMINE, FGF2, EGF, and ATP in a growth factor-free basal medium (BM) used for pluripotent stem-cell cultures^19^ (Fig. 1a). Moreover, only the RPCM containing all molecules was able to maintain simultaneously the proliferation and the expression of the key RPC transcription factors *VSX2, PAX6* and *RAX* (Supplementary Fig. 3). After 1 week in culture (W1), homogeneous and adherent hiRPCp0 still expressed VSX2, PAX6, and KI67 but not mCherry, as observed in W6 ROs (Fig. 1b and d, Supplementary Fig. 3). At W1, hiRPCp0 contained 50,2 ± 6,6% of KI67^+^ cells. RT-qPCR analysis confirmed EFTF expression and the positive selection of hiRPCs by the low expression of *CRX* (Fig. 1e). Moreover, pluripotency markers were shutdown in hiRPCs comparatively to hiPSCs (Supplementary Fig. 4). To bank retinal progenitors, hiRPCp0 were passed and expanded for 1 week in RPCM and hiRPCp1 were cryopreserved in stem cell-dedicated cryopreservation medium at 5 × 10^6^ cells/ml (Fig. 1a). Thus, this culture condition positively selected the hiRPCs which are the only mitotic cells at the expense of the the first differentiated post-mitotic cells. We investigated the possibility of passing thawed hiRPCs once a week to increase hiRPC production (Fig. 1f). One million thawed cells cultured in RPCM produced 4.6 ×10^6^ ± 0.6 hiRPCp2 in W1, 14.5 ×10^6^ hiRPCp3 in W2, and 43.23 ×10^6^ ± 2.96 in W3 (Fig. 1g). The mean multiplication factor between hiRPCp2 and hiRPCp4, was 3.6 ± 0.9 (Fig. 1g). Under RPCM culture conditions, the expression of EFTFs by W1-expanded hiRPCs was stable after passaging (Fig. 1h) and immunostaining confirmed the homogenous co-expression of VSX2, PAX6, KI67, and RAX in all cells (Fig. 1i, Supplementary Fig. 5). In addition, as expected, mCherry staining, which identifies CRX expression, was at background levels (Fig. 1i, Supplementary Fig. 5). Depending on the hiPSC line used to generate hiRPCs, EFTF expressions can decrease at passage 4 as observed in hiPSC-2-derived hiRPCs but without affecting their differentiation potential (Supplementary Fig. 6). After thawing, hiRPCs cultured in RPCM grew as bright cell clusters (Fig. 1i) and this morphology was lost for the neural phenotype when the cells are cultured in BM (Supplementary Fig. 7). Proliferation of hiRPCs was up to 10-fold higher in RPCM that in BM (Supplementary Fig. 7). Evidence for multipotency under RPCM culture conditions was shown by RT-qPCR, with enhanced expression of *RAX, PAX6, VSX2, SOX2*, and *SIX6* and the maintenance of *CCND1* expression, which sustains proliferation (Supplementary Fig. 7). On the contrary, in BM, EFTF and *CCND1* expression decreased, allowing hiRPC differentiation, highlighted by the upregulation of photoreceptor (*CRX*) and retinal ganglion cell (*BRN3A*) markers (Supplementary Fig. 7).

### Whole transcriptome analysis of hiRPCs

**Fig. 2:**
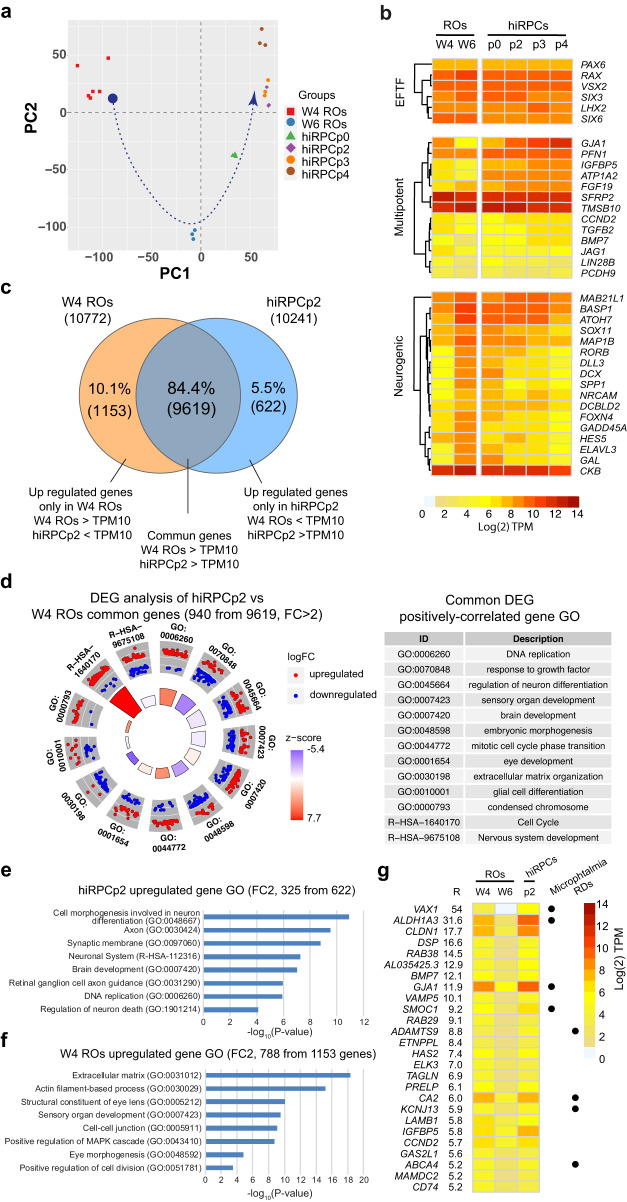
Transcriptomic characterization of hiRPCs by RNAseq analysis. **a** Principal component analysis (PCA) of W4 ROs, W6 ROs, hiRPCp0, and hiRPCp2–4. Each point represents one sample and biological replicates are shown in the same color. **b** Hierarchical clustering of EFTFs and multipotent and neurogenic gene markers in W4 ROS, W6 ROS, hiRPCp0, and hiRPCp2–4. hiRPCs were expanded for one week in RPCM. **c** Venn diagram of genes expressed in W4 ROs and hiRPCp2 with TPM ≥ 10. **d** Circular visualization (left panel) and table of the over-represented GO pathways of interest (right panel) identified with Metascape using the DEGs between hiRPCp2 and W4 ROs with a FC ≥ 2, FDR ≤ 0.05, and TPM ≥ 10 in both groups. Red dots (upregulated genes) and blue dots (downregulated genes) represent an overview of regulated genes in hiRPCp2 relative to W4 ROs. Z-score bars indicate whether an entire biological process is more likely to be increased or decreased based on the genes within it. **e** Gene ontology (GO) enrichment analysis for upregulated genes with fold change (FC) ≥ 2 in hiRPCp2. **f** GO enrichment analysis for upregulated genes with FC ≥ 2 and FDR ≤ 0.05 in W4 ROs. **g** Heatmap of genes with R = (W4 RO TPM) / (W6 RO TPM) > 5. Black dots indicate genes associated with microphthalmia or retinal dystrophies (RDs). hiPSC-5FC-derived cells.

We characterized the multipotency of the hiRPC line by comparing whole transcriptome sequencing (RNAseq analysis) of fresh hiRPCp0, thawed hiRPCp2, and passed hiRPCp3 and hiRPCp4 to W4 and W6 ROs. W4 ROs were mainly composed of RPCs and W6 ROs of both RPCs and RGCs (Supplementary Figs. 1 and 2). In total, 16,168 transcripts were detected (Supplementary Data 3) and those with a TPM expression value ≥ 10 were considered to be clearly expressed. This threshold was determined based on *CRX* expression in W4 ROs, when *CRX* was present at a very low level (2.8 TPM), whereas, in W6 ROs, *CRX* expression was higher, reflecting the progression of retinal differentiation (10.2 TPM) (Fig. 1c, Supplementary Data 3). Principal component analysis (PCA) showed four clusters based on the first principal component (PC1), illustrating a different transcriptional landscape between W4 ROs, W6 ROs, hiRPCp0, and other hiRPC passages (Fig. 2a). PC2 analysis supported the phenotypic reversion of the RPC population, composed of hiRPC clusters, to a transcriptome very close to that of early W4 ROs (Fig. 2a, bleu arrow). Heatmap analysis of EFTF expression confirmed high and stable expression of early retinal differentiation factors, such as *PAX6, RAX, VSX2, SIX3, SIX6*, and *LHX2*, in each sample (Fig. 2 b, top panel), as previously shown by RT-qPCR (Fig. 1h). We then focused on the expression of genes described to be involved in the multipotency or neurogenic property of retinal progenitors^20–22^ (Fig. 2b). As expected, the expression of multipotency markers tended to decrease in ROs between W4 and W6, whereas that of neurogenic genes increased, reflecting their early commitment to differentiation (Fig. 2b, middle panel). Nevertheless, the expression of these genes in RPCM-expanded hiRPCs was generally similar to the expression level observed in W4 ROs (Fig. 2b). This result is consistent with those of the PCA, confirming that the expanded RPC population corresponded to retinal progenitors at a similar progenitor stage as that found in W4 ROs. Remarkably, the expression of specific multipotency genes, such as *GJA1, PFN1, IGFBP5, ATP1A2*, and *FGF19*, increased under RPCM culture conditions, supporting the stemness characteristic of hiRPCs (Fig. 2b). The differential expression of selected multipotency genes (*GJA1, IGFBP5, ATP1A2, CCND2, BMP7*) and neurogenic genes (*BASP1, ATHO7, MAP1B, SPP1, FOXN4, GADD45A*) was confirmed by qRT-PCR (Supplementary Fig. 8). To better decipher the differences between RPCs in early ROs and expanded hiRPCs, we first compared the transcriptome of W4 ROs to that of hiRPCp2 to identify genes expressed by both groups or specifically by only one (Fig. 2c). These two groups showed 84.4% (9,619, Supplementary Data 4a) of their expressed genes to be in common. Based on our filtering criteria, 10.1% (1,153, Supplementary Data 4b) and 5.5% (622, Supplementary Data 4c) were considered to be specifically expressed in W4 ROs and hiRPCp2, respectively (Fig. 2c). Filtering of the differentially expressed genes (DEGs) between hiRPCp2 and W4 ROs with a fold change (FC) ≥ 2 or ≤ 2, a false discovery rate (FDR) ≤ 0.05, and minimum expression of 10 TPM led to the identification of 940 genes (Supplementary Data 4d). Pathway enrichment analysis of these DEGs and circular representation clearly showed the deregulation of pathways related to cell-cycle activity (GO:0006260; GO:0044772; GO:0000793; R − HSA − 1640170) and brain and eye development (GO:0007420; GO:0001654), with mostly upregulated genes, based on the calculated z-score. By contrast, the progression of W4 ROs toward hiRPCp2 occurred without promoting differentiation (GO:0070848; GO:0045664; GO:0007423; GO:0007423; GO:0048598; GO:0010001; R − HSA − 9675108), with the expression of most of the genes downregulated (Fig. 2d, Supplementary Data 4e). This analysis supports reinforcement of the multipotent and proliferative phenotype of hiRPCs cultivated in RPCM. We then filtered the results based on a FC ≥ 2 before Metascape analysis to focus the GO term enrichment on upregulated genes only in W4 ROs and hiRPCp2 (Fig. 2d). Thus, 325 genes were selected for hiRPCp2 (Fig. 2e, Supplementary Data 4f) and 788 for W4 ROs (Fig. 2f, Supplementary Data 4g), showing mainly variations in structural components of the cells that this could be a result of changing from free floating to adherent culture conditions.

### hiRPCs as a tool for retinogenesis studies

The maintenance of multipotency of the hiRPC population from W6 ROs during selection expansion in RPCM provides an innovative tool to trigger the expression of genes involved in the early stages of retinogenesis. We selected genes for which the expression differs (FC ≥ 2) in ROs between W4 and W6 and returns to their initial levels (as in W4 ROs) in expanded hiRPCp2 (Supplementary Fig. 8). Such an approach led to the identification of 617 restored genes, potentially representing actors involved in the maintenance of multipotency, as well as early neurogenesis (Supplementary Data 5a). To select genes potentially involved in the multipotency of RPCs, we ranked them by their expression ratio (R), representing fold-expression changes of genes between W4 and W6 ROs (Supplementary Data 5b). R > 1 and R < 1 identified genes for which the expression decreased or increased between W4 and W6 ROs, respectively. A heatmap representing genes with a ratio > 5 (Fig. 2g) confirmed the presence of genes already known to be multipotency makers, such as *GJA1, BMP7, IGFBP5*, and *CCND2*, validated by RT-qPCR (Supplementary Fig. 8). From these data, we propose a short list of genes (*VAX1, ALDH1A3, CLDN1, DSP, RAB38, VAMP5, SMOC1, RAB29, ADAMTS9, ETNPPL, HAS2, ELK3, TAGLN, PRELP, CA2, KCNJ13, LAMB1, GAS2L1, ABCA4, MAMDC2*, and *CD74*) and one long ncRNA (lncRNA, AL035425.3) that could be considered as potential new markers of RPC multipotency (Fig. 2g, Supplementary Data 5b). Interestingly, genes involved in microphthalmia (*VAX1, ALDH1A3, GJA1*, and *SMOC1*) and retinal dystrophies (*ADAMTS9, KCNJ13*, *CA2* and *ABCA4*) were also selected (Fig. 2g). Reciprocally, the R < 1 transcript list contained genes involved in neurogenesis, such as *MAP1B, SPP1, FOXN4*, and *GADD45A*, validated by RT-qPCR (Supplementary Fig. 8), and in the early differentiation of RPCs, such as *CRX, NEUROD1*, and *POU4F1* (Supplementary Data 5b).

### Neuroretinal cell production from hiRPCs

**Fig. 3:**
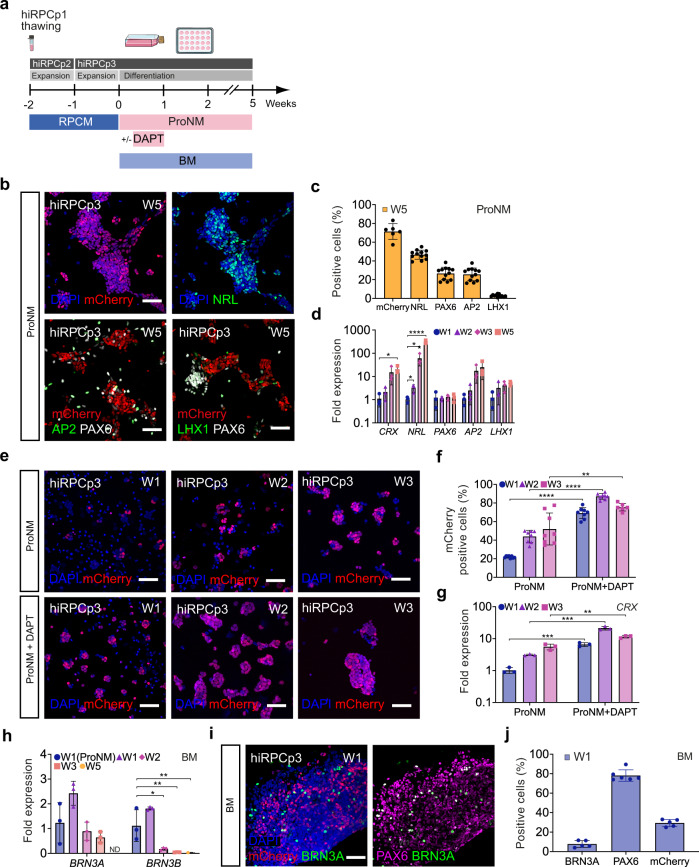
Differentiation of hiRPCs into neuroretinal cells. **a** Schematic diagram illustrating the differentiation protocol to generate early retinal cell types. **b** Immunofluorescence staining and endogenous expression of differentiated hiRPCp3 after five weeks (W5) of culture in ProNM for mCherry (hiPPCs), NRL (rod photoreceptor precursors), AP2 (amacrine cells), LHX1 (horizontal cells), and PAX6 (amacrine and horizontal cells). **c** High-content analysis of mCherry, NRL, AP2, and LHX1^+^ cells (%) in adherent cultures of hiRPCp3 differentiated in ProNM at W5. Data are presented as mean ± SD (n = 6 for mCherry and n = 12 for NRL, AP2 and LHX1). **d** RT-qPCR analysis of neuroretinal markers (*CRX, NRL, AP2, and LHX1*) in hiRPCp3 differentiated in ProNM from W1 to W3 and at W5. Data are normalized to that of hiRPCp3 at W1 and presented as mean ± SD (n = 3 per time point). **e** Endogenous expression of mCherry of hiRPCp3 differentiated in ProNM ± DAPT from W1 to W3. **f** High-content analysis of mCherry^+^ cells (%) in adherent cultures of hiRPCp3 differentiated in ProNM ± DAPT from W1 to W3. Data are presented as mean ± SD (n = 8 per time point). **g** RT-qPCR analysis of the PPC marker *CRX* in hiRPCp3 differentiated in ProNM ± DAPT from W1 to W3. Data were normalized to that of hiRPCp3 differentiated in ProNM at W1 and presented as mean ± SD (n = 3 per time point). **h** RT-qPCR analysis of the retinal ganglion cell (RGC) markers *BRN3A* and *BRN3B* in hiRPCp3 differentiated in ProNM or BM from W1 to W3 and W5. Data were normalized to that of hiRPCp3 differentiated in ProNM at W1 and presented as mean ± SD (n = 3 per time point). **i** Immunofluorescence staining of hiRPCp3 differentiated in BM at W1 for mCherry (hiPPCs), BRN3A (RGCs), and PAX6. **j** High-content analysis of mCherry (hiPPCs), BRN3A (RGCs), and PAX6^+^ cells (%) in adherent cultures of hiRPCp3 differentiated in BM at W1. Data are presented as mean ± SD (n = 5 for mCherry and BRN3A and n = 6 for PAX6). One-way ANOVA followed by a Dunnett’s multiple comparison test (**d**, **h**). Comparison to W1 (**d**) or W1 ProNM (**h**). Two-tailed Student’s t-test for two-group comparisons (**g**). ****p < 0,0001; ***p  <  0.001; **p  <  0.01; *p  <  0.05. Nuclei were counterstained with DAPI (blue). hiPSC-5FC-derived cells. Scale bar: **b**, **e**, **i**, 50 µm.

The next step was to demonstrate that our newly generated hiRPCs are able to differentiate into the various retinal cell types. Thus, proneural medium (ProNM) was used, as described for RO maturation in floating culture^18^, to trigger retinal differentiation (Fig. 1a). Although the differentiation of hiRPCs can be performed at different passages (Supplementary Figs. 9 and 10), we used here hiRPCp3 generated from thawed and expanded hiRPCp1 (Fig. 3a). After 5 weeks (W5) in culture, hiRPCs differentiated mainly into hiPPCs, identified by endogenous mCherry staining (71.4% ± 8.4), with among hiPPCs rod photoreceptor precursors co-expressing mCherry and NRL (46.7% ± 4.8) (Fig. 3b, c). Nonphotoreceptor cells expressing PAX6 represented 26.6% ± 6.7, corresponding to amacrine cells (PAX6^+^/AP2^+^, 25.7% ± 6.8) and horizontal cells (PAX6^+^/LHX1^+^, 2.9 ± 1.32) (Fig. 3b, c). RT-qPCR confirmed increased expression of these specific cell markers (*CRX*, *NRL*, *AP2*, and *LHX1*) during culture from W1 to W5 (Fig. 3d). To accelerate and promote hiPPCs generation, we cultured hiRPCs for 3 weeks in ProNM containing the NOTCH inhibitor DAPT, previously reported to promote photoreceptor differentiation within hiPSC-derived retinal organoids^7^ (Fig. 3a). Quantitative analysis showed mCherry^+^ hiPPCs to represent 69.0% ± 6.0 at W1, 87.2% ± 3.14 at W2, and 75.5% ± 4.1 at W3, whereas only 21.9% ± 1.2, 43.9 ± 6.56, and 52.0% ± 17.22 of mCherry^+^ cells were observed in the absence of DAPT at the same corresponding time points (Fig. 3f). RT-qPCR analysis confirmed the upregulation of the photoreceptor marker *CRX* in cultures with DAPT at W1, W2, and W3 (Fig. 3g). In ProMN culture condition, one million of hiRPCp3 generated 1,81.10^6^ ± 0,84, 2,67.10^6^ ± 1,09 or 3,58.10^6^ ± 0,72 derived retinal cells respectively at W1, W2, and W3. In parallel in ProMN + DAPT culture condition, one million of hiRPCp3 generated 2.31×10^6^ ± 1.12, 2.39×10^6^ ± 1,55 or 1.38×10^6^ ± 0,64 derived cells respectively at W1, W2, and W3. Similarly, differentiation during 1 week of the hiRPCp2 or hiRPCp4 generated from the nonfluorescent hiPSC-2 line showed higher hiPPCs population and *CRX* expression in the culture condition using the NOTCH inhibitor DAPT (Supplementary Fig. 10). Quantitative analysis at W1 showed CRX^+^ cells to represent 39.20% ± 4.27 (hiRPCp2) and 37.7% ± 9.32 (hiRPCp4) whereas only 17.2% ± 12.4 (hiRPCp2) and 23.6 ± 3.37 (hiRPCp4) of hiPPCs were observed in the absence of DAPT (Supplementary Fig. 10). To highlight the full competence of hiRPCs, we differentiated hiRPCs during 14 weeks on adherent condition (Supplementary Fig. 11). By immunochemistry, we identified the presence of emergent mature photoreceptors expressing RHODOPSIN, BLUE OPSIN (OPN1SW) and Red/Green OPSIN (OPN1MLW), bipolar cells (VSX2/PRKCA) and Müller glia cells (GS/SOX9). We confirmed by RT-qPCR the expression of the specific genes *CRX, NRL, ARR3, OPN1SW, RHO, AP2, RLBP1, PRKCA* targeting these retinal cell types (Supplementary Fig. 11). Concerning the differentiation of hiRPCs into the RGC lineage, RT-qPCR analysis of *BRN3A* and *BRN3B* expression and immunostaining of BRN3A showed that ProNM and BM can induce the generation of RGCs from hiRPCs (Fig. 3h–j, Supplementary Figs. 9 and 10). However, culturing hiRPCs in BM allowed higher expression of RGC markers at W1 relative to their culture in ProNM (Fig. 3h). Decreased BRN3A and BRN3B expression after W1 may reflect the loss of hiRPC-derived RGCs under these culture conditions (Fig. 3h). Quantitative analysis of identified RGCs by immunostaining in BM at W1 showed 7.7% ± 3.5 BRN3A^+^ cells among 78.1% ± 5.9 of PAX6^+^ cells in the presence of 29.4% ± 3.6 of mCherry^+^ hiPPCs (Fig. 3i, j).

### Retinal pigmented epithelial cell generation from hiRPCs

**Fig. 4:**
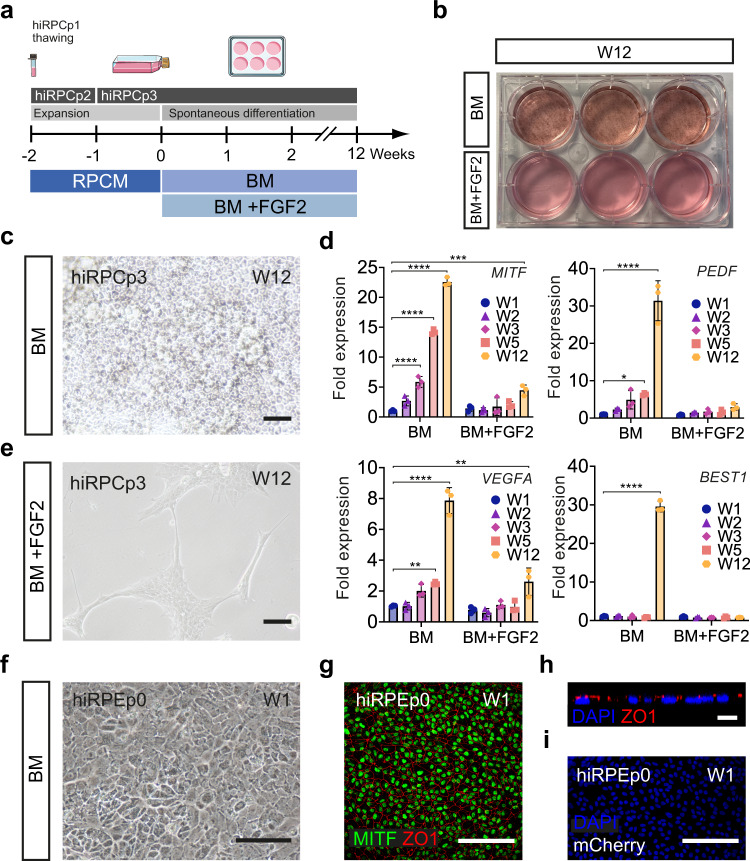
Differentiation of hiRPCs into RPE cells **a** Schematic diagram illustrating the differentiation protocol of RPE generation from hiRPCp3. **b** Photograph of a six-well plate of differentiated hiRPCp3 at W12 using BM ± FGF2. **c** Phase-contrast image of hiRPCp3 cultured in BM at W12. **d** RT-qPCR analysis of specific RPE markers in hiRPCp3 cultured in BM or BM + FGF2 from W1 to W3 and at W5 and W12. All expression was normalized to that of W1 hiRPCs cultured in BM. Data are presented as mean ± SD (n = 3 per time point). **e** Phase-contrast image of hiRPCp3 at W12 cultured in BM + FGF2. **f** Phase-contrast image of hiRPC-derived RPE at passage 0 (hiRPEp0) at W1. **g** immunofluorescence staining of hiRPEp0 for MITF, ZO1 at W1. **h** Z-stack confocal image of the apical ZO1 marker on hiRPEp0 at W1. **i** Endogenous mCherry staining in hiRPEp0 at W1. One-way ANOVA followed by a Dunnett’s multiple comparison test (**c**, **e**, **g**, **h**). Comparison to W1. ****p < 0,0001; ***p  <  0.001; **p  <  0.01; *p  <  0.05. Nuclei were counterstained with DAPI (blue). hiPSC-5FC-derived cells. Scale bar: **c**, **e**, **g**, **I**, 50 µm. **h**, 10 µm.

Based on the identification of the preneurogenic state of hiRPCs, we tested the ability of these new progenitors to differentiate into RPE cells. hiRPCs were submitted to spontaneous differentiation using BM for 12 weeks (W12) (Fig. 4a). Under these conditions, pigmented cells could be observed (Fig. 4b) and phase contrast imaging confirmed the RPE cell morphology (Fig. 4c). RT-qPCR analysis for specific RPE markers showed increased expression of *MITF*, *PEDF*, *VEGFA*, and *BEST1* from W1 to W12, confirming cell commitment towards the RPE lineage (Fig. 4d). To exclude the potential contamination of RPE cells in banked hiRPCs, we added FGF2, reported to be a factor necessary for the differentiation of retinal progenitors towards the neuroretinal lineage, at the expense of the RPE lineage, during retinogenesis^23^. The presence of FGF2 completely prevented the appearance of RPE cells and markers (Fig. 4b, d, e). HiRPC-derived RPE (hiRPE) cells at W12 were passed (noted as hiRPEp0) and formed a confluent cell monolayer after 1 week that displayed the classical RPE morphology (Fig. 4f). Immunostaining targeting MITF and ZO-1 confirmed the identity of RPE cells, with regular apical tight junctions (Fig. 4g, h) and without mCherry expression (Fig. 4i).

## Discussion

**Fig. 5:**
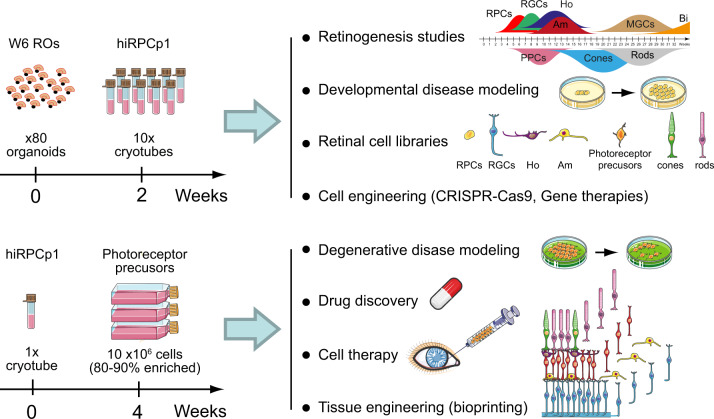
Usefulness of hiRPCs and their cell derivatives. hiRPCs could be used for retinogenesis studies, developmental disease modeling, the generation of retinal cell libraries, and as cell substrates for cell engineering. Derived retinal cells from hiRPCs could be used for patient-based degenerative disease modeling, drug discovery, cell therapy, and tissue engineering.

In this study, we demonstrate that RPCs within hiPSC-derived ROs can be isolated and cryo-preserved after steps of expansion in adherent culture conditions. Banked hiRPCs maintained their multipotency illustrated by their ability to differentiate into all retinal cell types. The multipotency of the hiRPCs was maintained by culture conditions using a RPC-dedicated medium containing specific factors to mimic the environment of retinogenesis. During retinal development, RPCs have to maintain the proper balance between proliferation and differentiation to produce the full range of retinal cell types in sufficient numbers to generate a complete retina^24^. It is well established that cell fate determination is controlled by a combination of extrinsic cues and intrinsic factors^24–26^. Based on these data, various approaches, focusing mainly on extracellular signals, have been developed to isolate multipotent RPCs and amplify them. However, these processes failed to maintain multipotency in expanded RPCs^12–14^. Based on the literature, we hypothesized that modulating specific signaling pathways, such as WNT, SHH, EGF, and FGF signaling, could be sufficient to act on the intrinsic factors that control the proliferation and multipotency of RPCs. To recapitulate the environment of the developing tissue, we selected five extrinsic factors: the GSK3 inhibitor CHIR99021, mimicking WNT activation through beta-catenin accumulation in the cytoplasm and maintaining the human stem cell fate and expression of retinal progenitor markers;^27,28^ PURMORPHAMINE as an activator of the Shh pathway, sustaining RPC self-renewal and multiplication^26,29–33^ and reproducing Shh pathway activation by ganglion cells (RGCs) during retinogenesis; FGF2, the most highly used factor to maintain the pluripotency of human embryonic stem cells and iPSCs in culture and shown to also potentiate the action of the Shh pathway and avoid RPE differentiation of progenitors^34,35^; EGF, classically coupled to FGF2 in neural stem cell culture and promoting RPC proliferation at the expense of their differentiation^36^; and ATP, which controls the cell cycle and RPC proliferation in the developing retina^37–40^, mimicking RPE-release of ATP into the subretinal space^37^. Thus, we demonstrate that simultaneous activation of these specific pathways allows the selection and amplification of hiRPCs. Transcriptomic analysis (RT-qPCR and RNAseq) and immunofluorescence staining confirmed the high similarity between RPCs within ROs and hiRPCs thawed and expanded until passage 4. The proneurogenic phenotype of hiRPCs was highlighted by transcriptomic analysis, as well as by the differentiation capacity of the hiRPCs to differentiate into all neural retinal cell types including mature photoreceptors. However, although at W14 few differentiated photoreceptors expressed mature markers as RHODOPSIN, BLUE OPSIN or RED/GREEN OPSIN, these expressions were consistent with the retinogenesis described in ROs at this time^16^. Interestingly, in addition to the ability to differentiate into neuroretinal cells and muller glial cells, hiRPCs can also be differentiated into RPE cells. This shows that RPCs within ROs still possess the intrinsic ability to regain the preneurogenic phenotype, depending on the extrinsic environment. This valuable multipotent cell line was easy to use and produced millions of differentiated retinal cells in a short time under adherent conditions from a cryopreserved cell stock. From one banked tube of hiRPCp1, more than 10 millions of hiPPCs can be produced within enriched cultures of up to 90% in 4 weeks and without a purification step. This scale-up production of hiPPCs should be useful for disease modeling, tissue engineering and drug discovery (Fig. 5). This potential source of transplantation-compatible cell population^41–43^ was produced under xeno-free conditions as needed for future cell therapies but the in vivo maturation capabilities of the hiPPCs still need to be evaluated at this stage. Moreover, we showed differences in the differentiation ability among lines. This observation could indicate that the treatment with molecules, as the NOTCH inhibitor DAPT, must to be adapted to obtain optimal number of expected cells. Similarly, the ability to generate RGCs from hiRPCs may pave the way towards the future treatment of diseases that affect the optical nerve, such as glaucoma or diabetic retinopathy. In addition to being a substrate for the scaling up of the production of retinal cell types, hiRPCs appear to be a useful tool to study retinogenesis and associated diseases. Indeed, transcriptomic analysis comparing RO and hiRPC gene expression revealed potential markers of multipotency, neurogenesis, and differentiation. Of note, specific transcriptomic analysis using gene expression filters selected genes involved in developmental retinal diseases, such as microphthalmia (*VAX1*^44^, *ALDH1A3*^45^, *GJA1*^46^, *SMOC1*^47^) and retinal dystrophies, such as age-related macular dystrophy (*ADAMTS9*^48,49^), Leber congenital amaurosis (*KCNJ13*^50,51^), glaucoma (*CA2*^52^), and Stargardt disease (*ABCA4*^53^), supporting the use of this workflow to identify new genes that cause retinal diseases. Overall, we used an innovative culture condition to generate a human retinal multipotent cell line. Although future experiments are needed to evaluate all applications using hiRPCs and its derivatives, this advanced cell line could become a gold standard cellular substrate to study retinogenesis, create disease models, and generate retinal cell libraries useful for cell therapy and drug discovery (Fig. 5). Thus, bankable hiRPCs could convert hope to reality for the future accessibility of innovative treatments for millions of people suffering from degenerative retinal diseases.

## Methods

### Human subjects

Postmortem eye tissues used to generate the hiPSC-5F^17^ and hiPSC-5FC^15^ clones were collected in accordance with the French bioethics law at the Laboratory of Anatomy of the Faculty of Medicine of St-Etienne, France. Handling of donor tissues adhered to the tenets of the Declaration of Helsinki of 1975 and its 1983 revision in protecting donor confidentiality. Skin biopsy used to generate the hiPSC-2 clone^7,16^ were obtained from informed patient under the approval of French regulatory agencies.

### Human iPSC culture

Experiments were conducted using the human fluorescent reporter AAVS1::CrxP_H2BmCherry-hiPSC line (hiPSC-5FC line), allowing the identification of photoreceptor lineage-committed cells by endogenous mCherry staining^15^ and with the nonfluorescent hiPSC-5F^17^ and the hiPSC-2 lines^7,16^. HiPSCs were cultured under feeder-free conditions on truncated recombinant human vitronectin rhVTN-N (STEMCELL Technologies)-coated dishes in mTeSR^TM^1 Medium (STEMCELL Technologies). Cells were routinely cultured in 6-cm^2^ dishes at 37 °C in a standard 5% CO_2_/95% air incubator with a daily medium change. HiPSCs were passaged weekly, as previously described, using 2 mL enzyme-free gentle cell-dissociation reagent (STEMCELL Technologies) for 6 min at room temperature^16^. For immunostaining, hiPSCs were passaged and replated in 24 well-plates containing glass coverslips precoated with human vitronectin rhVTN-N (STEMCELL Technologies). Cells were returned to the incubator at 37 °C and 5% C02 for 1 week and then fixed with 4% PFA in PBS for 10 min.

### Retinal organoid differentiation

RO generation was based on our previously established adherent hiPSC differentiation protocol^7,16,18^. HiPSCs were expanded to 70 to 80% confluence in 6-cm diameter dishes as described above. At this time, defined as day 0 (D0), hiPSCs were cultured in chemically defined Essential 6 (E6) medium with 10 units/ml penicillin and 10 mg/ml streptomycin (Thermo Fisher Scientific). At D2, cells were switched to E6N2 medium, composed of E6 medium, 1% N2 supplement, 10 units/ml penicillin, and 10 mg/ml streptomycin (Thermo Fisher Scientific). Media was changed every 2 to 3 days. After 4 weeks (W4), identified self-formed retinal organoids were isolated using a needle and cultured in six-well-plates (10–15 organoids per well) as floating structures in proneural medium (ProNM) supplemented with 10 ng/ml animal-free recombinant human FGF2 (Peprotech) and half of the medium was changed every 2 to 3 days. ProNM is composed of chemically defined DMEM:Nutrient Mixture F-12 (DMEM/F12, 1:1, L-Glutamine), 1% MEM nonessential amino acids, 2% B27 supplement (Thermo Fisher Scientific), 10 units/ml penicillin, and 10 mg/ml streptomycin. After 1 week, FGF2 was removed from the ProNM and half of the medium was changed every 2- to 3 days.

### Isolation, expansion, and cryopreservation of hiRPCs

HiRPCs were isolated from 6-week-old (W6) ROs. Before dissociation, the surrounding pigmented portion of the ROs was discarded under a stereomicroscope. Between 70 to 90 structures were washed twice in Ringer’s solution (155 mM NaCl, 5 mM KCl, 2 mM CaCl_2_, 1 mM MgCl_2_, 2 mM NaH_2_PO_4_, 10 mM HEPES, and 10 mM glucose) and enzymatically dissociated using two units of papain (Worthington, WOLS3126), previously activated in SAP solution (125 mM NaCl, 3.6 mM KCl, 1.18 mM MgCl_2_, 22.6 mM NaHCO_3_, 0.02 mM NaH_2_PO_4_, 0.028 mM Na_2_HPO_4_, 1.2 mM Na_2_SO_4_, 10 mM glucose; 0.54 mM Na_2_EDTA), for 30 min at 37 °C. ROs were dissociated by up and down pipetting in the presence of 1 μg/ml DNAse (Sigma-Aldrich), preventing cell aggregation. After complete dissociation, the papain was inactivated with prewarmed ProNM. Cells were centrifuged and resuspended in prewarmed RPCM medium. RPCM is composed of E6 medium, 10 units/ml penicillin and 10 mg/ml streptomycin (Thermo Fisher Scientific), 3 µM CHIR99021 (Euromedex), 1 µM Purmorphamine (Euromedex), 10 ng/mL FGF2 (Peprotech), 100 ng/mL EGF (Peprotech), and 100 µM ATP (Sigma-Aldrich). Cells were plated at a density of 3 ×10^5^ cells/cm² onto T25-cm^2^ dishes previously coated with Geltrex^TM^ (Thermo Fisher Scientific). At this time, hiRPCs were designated as being at passage 0 (hiRPCp0). Cells were incubated at 37 °C in a standard 5% CO_2_/95% air incubator and the medium was changed every 2 to 3 days for 1 week. At 70 to 80% confluency, hiRPCp0 cultured in RPCM were dissociated using 2 mL TrypLE^TM^ Express (Gibco) per T25-cm^2^ for 8 min at 37 °C and the reaction stopped by dilution with 8 mL prewarmed RPCM. HiRPCs were centrifuged at 110 × g for 3 min and resuspended in prewarmed RPCM. Cell proliferation was measured using an automated cell counter (Scepter 3.0 Handheld Automated Cell Counter, Millipore). Cells were seeded in Geltrex^TM^ (Thermo Fisher Scientific) precoated T25-cm^2^ plates at a density of 1.5 ×10^5^ cells/cm² and designated as hiRPCs at passage 1 (hiRPCp1). Cells were incubated at 37 °C in a standard 5% CO_2_/95% air incubator and the medium was changed every 2 to 3 days for 1 week. At 70%–80% confluency, hiRPCp1 were dissociated using TrypLE^TM^ Express (Gibco) and cryopreserved at 5 ×10^6^ cells/ml using Cryostor® cryopreservation medium (STEMCELL Technologies) in cryogenic tubes. Tubes were placed in an isopropanol-based freezing container at −80 °C for a minimum of 4 h and kept in a −150 °C freezer for long-term storage.

### Molecular cocktail testing

Experiments were conducted using derived cells from the human fluorescent reporter hiPSC-5FC line and the hiPSC-2 line. After complete ROs dissociation, cells were centrifuged and resuspended in 6 different culture conditions: E6 medium; E6 medium and 1 µM Purmorphamine; E6 medium and 3 µM CHIR99021; E6 medium and 100 µM ATP; E6 medium, 1 µM Purmorphamine, 3 µM CHIR99021, 100 µM ATP or RPCM medium. For immunostaining, hiRPCp0 were replated at a density of 3 ×10^5^ cells/cm² in 24 well-plates containing glass coverslips with a glass bottom (Celvis) precoated with Geltrex^TM^ (Thermo Fisher Scientific). Cells were returned to the incubator at 37 °C and 5% C02 for 1 week and then fixed with 4% PFA in PBS for 10 min.

### Differentiation of hiRPCs

The majority of differentiation experiments were performed using hiRPCp3 from banked hiRPCp1. Thawed hiRPCp1 were seeded at 5 ×10^4^ cells/cm² (noted hiRPCp2) using Geltrex® (Thermo Fisher Scientific) precoated T25-cm^2^ dishes or six-well plates. After 1 week under expansion conditions using RPCM, hiRPCp2 were passaged and experiments performed using 1-week expanded hiRPCp3. Spontaneous differentiation was performed using a basal medium (BM) composed of E6 medium, 10 units/ml penicillin, and 10 mg/ml streptomycin (Thermo Fisher Scientific). Directed differentiation was performed using ProNM (i.e., retinal differentiation). Cell culture to promote hiPPCs was performed using ProNM supplemented with the Notch Inhibitor DAPT at 10 μM from day 2 to day 7 of the 3 weeks of differentiation. For immunostaining and high-content image quantification at the end of differentiation, hiRPC-derived retinal cells were enzymatically dissociated using 0.24 units/cm^2^ papain as previously described^16^. Cells were replated at 1.5 ×10^5^ cells/cm^2^ in 24 well-plates containing glass coverslips or in 96-well plates with a glass bottom (Celvis) precoated with Poly-D-lysine (2 µg/cm², Merk) and laminin (1 µg/cm², Sigma-Aldrich). Cells were returned to the incubator at 37 °C and 5% C0^2^ for 24 h and then fixed with 4% PFA in PBS for 10 min. For long-term differentiation experiments, hiRPCp2 (hiPSC-5F line) were cultured in ProNM during 14 weeks supplemented with FGF2 in the first 2 weeks. At W7, hiRPC-derived retinal cells were enzymatically dissociated using papain as previously described and replated at 1.5 ×10^5^ cells/cm^2^ in 6-well plates precoated with Poly-D-lysine (2 µg/cm², Merk) and laminin (1 µg/cm², Sigma-Aldrich) allowing the culture differentiation up to W14. Cells were incubated at 37 °C in a standard 5% CO_2_/95% air incubator and the medium was changed every 2 to 3 days.

### RNA extraction and Taqman assay

Total RNA was extracted using a Nucleospin RNA II kit (Macherey-Nagel) according to the manufacturer’s protocol and RNA yields and quality assessed using a NanoDrop spectrophotometer (Thermo Fisher Scientific). cDNA was synthesized from 100 ng total RNA using the QuantiTect reverse transcription kit (Qiagen) following the manufacturer’s recommendations. Synthesized cDNA was then diluted 1/20 in DNase-free water before performing real-time quantitative PCR (RT-qPCR). RT-qPCR analysis was performed using an Applied Biosystems real-time PCR device (7500 Fast System) with custom TaqMan® Array 96-Well Fast plates and TaqMan® Gene expression Master Mix (Life Technologies) following the manufacturer’s instructions. All primers and MGB probes labeled with FAM for amplification were purchased from Life Technologies (Supplementary Data 1). Results were normalized against those obtained with 18 S rRNA and the quantification of gene expression was based on the delta-deltaCt method (Eq. 1) in three minimum independent biological experiments.

1 $${2}^{-{{{{{\rm{ddCt}}}}}}}\,{{{{{\rm{with}}}}}}\,{{{{{\rm{ddCt}}}}}}={[{({{{{{{\rm{Ct}}}}}}}_{{{{{{\rm{gene1}}}}}}}-{{{{{{\rm{Ct}}}}}}}_{18{{{{{\rm{S}}}}}}})}_{{{{{{\rm{t2}}}}}}}]}_{{{{{{\rm{n}}}}}}}-{[{({{{{{{\rm{Ct}}}}}}}_{{{{{{\rm{gene1}}}}}}}{-{{{{{\rm{Ct}}}}}}}_{{{{{{\rm{18S}}}}}}})}_{{{{{{\rm{t1}}}}}}}]}_{{{{{{\rm{n}}}}}}}$$

where Ct = cycle threshold; t = time; n = independent biological experiments.

### RNAseq analysis

RNA-seq libraries were constructed by Integragen Genomics® from 400 ng total RNA (RIN > 7) using NEBNext Ultra II Directional RNA (New England Biolabs) and paired-end sequencing of 100 base-pair fragments was performed on a Novaseq 6000 system (Illumina). Image analysis and base calling was performed using an Illumina Real Time Analysis (3.4.4) device with default parameters. Pass-filtered reads were mapped using STAR 2.7.3a^54^ and aligned to Ensembl genome assembly GRCh38 (release 98). This annotation includes cDNA, miRNA, long noncoding RNA, pseudogene, and gene predictions. For differential expression analysis, a count table of the gene features was obtained using FeatureCounts^55^. For gene level analysis, EdgeR was used for normalization, differential expression analysis, and to compute TPM (transcripts per million) values^56^. Comprehensive gene-list analysis, enriched biological pathways, and gene annotation from differential expression were based on the Gene Ontology (GO) classification system using Metascape^57^. R packages were used for data mining, including GOPlot for pathway data graphical representation^58^. GEO accession: GSE220792.

### High-content Image analysis

For high-content quantitative analysis, images were acquired using an automated microscope (Arrayscan VTI HCS Reader, Thermo Fisher Scientific) of the screening facility. To count the number of hiPPCs expressing mCherry (red) or immunofluorescence-stained cells, an image analysis workflow was created using HCS Studio Cell Analysis Software (Thermo Fisher Scientific) with the TargetActivation BioApplication. Image analysis included an image preprocessing step followed by a segmentation step, allowing classification of the pixels and objects based on fluorescence intensity thresholds. High-content quantitative analysis of hiPPCs derived from hiPSC-2 line were acquired using the automated microscope CQ1 (Confocal Quantitative Image Cytometer, Yokogawa) » and analyze in by the CellpathFinder Software (Yokogawa).

### Immunostaining of ROs, hiRPCs, and hiRPC-derived cells

RO sections or retinal cells were fixed with 4% PFA in PBS for 10 min before immunostaining. After washing with PBS, nonspecific binding sites were blocked for 1 h at room temperature with PBS containing 0.2% gelatin and 0.25% Triton X-100 (blocking buffer) and then overnight at 4 °C with the primary antibody (Supplementary Data 2) diluted in blocking buffer. Samples were washed four times for 5 min in PBS with 0.1% Tween and then incubated for 1 h at room temperature with either AlexaFluor 488 or 647 secondary antibodies (Interchim) diluted at 1:600 in blocking buffer. Cells were washed two times for 5 min in PBS with 0.1% Tween and once for 5 min in PBS with 0.1% Tween and DAPI (4’,6-diamidino-2-phenylindole) at 1:1000. Samples were washed with PBS and mounted on slides for imaging. Fluorescence was captured using an Olympus FV1000 confocal microscope equipped with 405, 488, 543, and 633 nm lasers. Images were acquired using a 1.55 or 0.46 µm step size and corresponded to the projection of 20 to 40 optical sections.

### Statistics and reproducibility

Statistical analyses represent the mean of at least three independent experiments. Data were averaged and are expressed as means ± SDs (Standard Deviation scores). Statistical analysis was performed using Prism 9 (GraphPad software) with appropriate statistical tests. A two-tailed Student’s t-test was carried out for two-group comparisons, and ordinary one-way analysis of variance (ANOVA) followed by a Dunnett’s test was performed for multiple-group comparisons. Values of p < 0.05 were considered statistically significant.

## Supplementary information

Editorial Assessment Report

Supplementary Information

Supplementary Data 1

Supplementary Data 2

Supplementary Data 3

Supplementary Data 4

Supplementary Data 5

Supplementary Data 6

## Data Availability

The raw RNA sequencing data are deposited in the Gene Expression Omnibus (GEO) database under accession code GSE220792. Data underlying the graphs presented in the figures and supplementary figures are available in Supplementary Data 6. Additional data inquiries could be addressed to the corresponding author Sacha Reichman (sacha.reichman@inserm.fr).
