# Supplementary material for: Bankable human iPSC-derived retinal progenitors represent a valuable source of multipotent cells
Source: Commun Biol. 2023 Jul 21;6:762. doi: 10.1038/s42003-023-04956-2 (PMC10362027; doi:10.1038/s42003-023-04956-2)
Supplement: Supplementary file 1 — Editorial Assessment Report [file 42003_2023_4956_MOESM1_ESM.pdf]

nature portfolio

GUIDED OPEN ACCESS

nature methods | nature communications | communications biology

Contents of this report

1. **Manuscript details**: overview of your manuscript and the editorial team.
2. **Review synthesis**: summary of the reviewer reports provided by the editors.
3. **Editorial recommendation**: personalized evaluation and recommendation from all 3 journals.
4. **Annotated reviewer comments**: the referee reports with comments from the editors.
5. **Open research evaluation**: advice for adhering to best reproducibility practices.

About the editorial process

Because you selected the **Nature Portfolio Guided Open Access** option, your manuscript was assessed for suitability in three of our titles publishing high-quality work across the spectrum of methods research: ***Nature Methods***, ***Nature Communications***, and ***Communications Biology***. More information about Guided Open Access can be found here.

Collaborative editorial assessment

Your editorial team discussed the manuscript to determine its suitability for the Nature Portfolio Guided OA pilot. Our assessment of your manuscript takes into account several factors, including whether the work meets the **technical standard** of the Nature Portfolio and whether the findings are of **immediate significance** to the readership of at least one of the participating journals in the Nature Portfolio Guided Open Access methods cluster.

Peer review

Experts were asked to evaluate the following aspects of your manuscript:

- **Novelty** in comparison to prior publications;
- **Likely audience** of researchers in terms of broad fields of study and size;
- **Potential impact** of the study on the immediate or wider research field;
- **Evidence** for the claims and whether additional experiments or analyses could feasibly strengthen the evidence;
- **Methodological detail** and whether the manuscript is reproducible as written;
- Appropriateness of the **literature review**.

Editorial evaluation of reviews

Your editorial team discussed the potential suitability of your manuscript for each of the participating journals. They then discussed the revisions necessary in order for the work to be published, keeping each journal's specific editorial criteria in mind.

Journals in the Nature portfolio will support authors wishing to transfer their reviews and (where reviewers agree) the reviewers' identities to journals outside of Springer Nature.
If you have any questions about review portability, please contact our editorial office at guidedoa@nature.com.

Manuscript details

Tracking number	Submission date	Decision date	Peer review type
GUIDEDOA-22-00427	Feb 21, 2022	Mar 31, 2022	Single-blind

Manuscript title

Bankable Human iPSC-Derived Retinal Progenitors: A New Source of Multipotent Cells

Author details

Sacha Reichman

Affiliation: Sorbonne Université, INSERM, CNRS, Institut de la Vision

Editorial assessment team

**Primary editor**	**George Inglis**<br>Home journal: ***Communications Biology***<br>ORCID: **0000-0002-9069-5242**<br>Email: **george.inglis@us.nature.com**
**Other editors consulted**	**Madhura Mukhopadhyay**<br>Home journal: ***Nature Methods***<br>ORCID: **0000-0003-3907-3955**<br><br>**Cara Eldridge**<br>Home journal: ***Nature Communications***<br>ORCID: **0000-0001-7001-2312**
**About your primary editor**	George received his PhD in Genetics and Molecular Biology from Emory University, where he studied mouse models of voltage-gated sodium channel dysfunction and epilepsy. He also has research experience in epigenomics and *in vitro* models of neuronal development. George joined the editorial team of *Communications Biology* in September 2020 and is based in the New York office.

Editorial assessment and review synthesis

**Editor's summary and assessment**	Retinal progenitor cells (RPCs) have historically been difficult to isolate and expand without sacrificing their potential to differentiate into various retinal cell types. Here, the authors describe a differentiation protocol that allows for efficient generation of RPCs while still maintaining an efficient differentiation potential. Their protocol relies on first generating iPSC-derived retinal organoids (4-6 weeks), then isolating RPCs, which can then be expanded in RPCM prior to cryopreservation. They demonstrate that these RPCs continue to grow efficiently after 4 passages, and maintain expression of multipotency markers, as verified by IHC, RT-PCR, and RNA-seq, and as demonstrate that RPCs could then be differentiated into photoreceptor precursor cells, as well as retinal pigmented epithelial cells. Altogether, they state that their approach would be useful in generating additional retinal subtypes and identifying factors linked to retinogenesis or retinal diseases.<br><br>The editors jointly decided to send this manuscript out to review based on the potential utility of this method in generating multiple retinal cell types. However, the fact that several differentiation protocols already existed (and were not benchmarked) in this study, and the lack of functional followup on candidate retinogenesis or multipotency genes, prohibited further consideration by *Nature Methods*. Similarly, the editors at *Nature Communications* had some concerns about the level of advance over existing protocols, and the limited validation of the current method using a single cell line.
**Editorial synthesis of reviewer reports**	While the reviewers found the topic to be of some interest, they shared several concerns regarding the novelty of the method, its reproducibility (given the reliance on a single cell line), and the molecular characterization of the various retinal cell types.<br><br>Given these concerns, *Nature Methods* and *Nature Communications* are unable to invite a revision. However, *Communications Biology* would be interested in considering a manuscript that (at a minimum) includes the following revisions:<br>1. Please repeat the differentiation protocol, staining/expression analyses (apart from RNA-seq) on at least one other cell line, given concerns about reproducibility from Reviewers #2-3.<br>2. Please include additional staining for relevant multipotency or mature markers as outlined by all reviewers. On a related note, it would be necessary either to further investigate the potential of these cells to differentiate into other cell types (cone, MG, etc.) as outlined by Reviewer #1, or expand on the limitations of this current approach as outlined by Reviewer #3. It would also be helpful to include additional metrics, like proliferation rate, as noted by Reviewer #3.<br>3. Please justify the experimental setup, including the rationale for doses and inclusion of the extrinsic factors, as noted by Reviewers #1 and #4. It would also be necessary to expand the Introduction and Discussion to clarify any distinguishing features of this method, compared to existing alternatives.

Editorial recommendation

Nature Methods<br><br>Revision not invited	In the absence of extensive benchmarking against existing similar methods and lack of a clear technical advance, we do not think that the method presented will have a sufficiently significant and immediate impact on a broader readership to justify publication in *Nature Methods*.
Nature Communications<br><br>Revision not invited	*Nature Communications* has concerns about the lack of sufficient benchmarking against existing methods, and finds that the novelty of the method does not meet our editorial bar for further consideration at this stage. We also find that the technical evaluation is too limited for us to consider this further.
Communications Biology<br><br>Major revisions with extension of the work	*Communications Biology* would be interested in considering a revised manuscript that (at a minimum) includes at least one other cell line, shows additional staining for relevant multipotency or mature cell markers, investigates the potential for derived RPCs to differentiate into other cell types (or, further outlines limitations), justifies the differentiation strategy and underlying rationale, and expands on the novelty of this method compared to existing alternatives.

Next steps

**Editorial recommendation:**	Our top recommendation is to revise and resubmit your manuscript to *Communications Biology.* We feel the additional experiments required are reasonable to achieve within a six-month time frame.
**Note**	As stated on the previous page, *Nature Methods* and *Nature Communications* are not inviting a revision at this time. Please keep in mind that the journal will not be able to consider any appeals of their decision through Guided Open Access.

Revision

To follow our recommendation, please upload the revised manuscript files using **the link provided in the decision letter**. Should you need assistance with our manuscript tracking system, please contact Adam Lipkin, our Nature Portfolio Guided OA support specialist, at guidedOA@nature.com.

Revision checklist

- ☐ Cover letter, stating to which journal you are submitting
- ☐ Revised manuscript
- ☐ Point-by-point response to reviews
- ☐ Updated Reporting Summary and Editorial Policy Checklist
- ☐ Supplementary materials (if applicable)

Submission elsewhere

If you choose not to follow our recommendations, you can still take the reviewer reports with you.

Option 1: Transfer to another Nature Portfolio journal
Springer Nature provides authors with the ability to transfer a manuscript within the Nature Portfolio, without the author having to upload the manuscript data again. To use this service, **please follow the transfer link provided in the decision letter.** If no link was provided, please contact guidedOA@nature.com.

> *Note that any decision to opt in to In Review at the original journal is not sent to the receiving journal on transfer. You can opt in to In Review at receiving journals that support this service by choosing to modify your manuscript on transfer.*

Option 2: Portable Peer Review option for submission to a journal outside of Nature Portfolio
If you choose to submit your revised manuscript to a journal at another publisher, we can share the reviews with another journal outside of the Nature Portfolio if requested. You will need to request that the receiving journal office contacts us at guidedOA@nature.com. We have included editorial guidance below in the reviewer reports and open research evaluation to aid in revising the manuscript for publication elsewhere.

Annotated reviewer reports

The editors have included some additional comments on specific points raised by the reviewers below, to clarify requirements for publication in the recommended journal(s). However, please note that all points should be addressed in a revision, even if an editor has not specifically commented on them.

Reviewer #1 information	
**Expertise**	This reviewer has expertise in retinal development and repair.
**Editor's comments**	This reviewer outlines the need to describe the potential advance of this method over existing alternatives, and clarify the setup of the differentiation tool. They also emphasize the need to further validate the differentiation potential of any RPCs derived from this method.

Reviewer #1 comments	
**Section**	**Annotated Reviewer Comments**
**Remarks to the Author: Impact**	The authors have established an in vitro method to expand and bank retinal progenitor cells from hiPSC-derived retinal organoids. These cell lines can passage, be cryo-recovered and maintain expression of a number of transcripts associated with retina and neural progenitor multipotency. Upon transfer to differentiation conditions (primarily mitogen withdrawal) these RPCs induce expression of photoreceptor reporters (Crx- mcherry) and other lineage markers consistent with RGC, amacrine and horizontal cells.<br><br>Establishing a biobank-compatible source of RPCs for lineage differentiation (primarily photoreceptors) would be a significant advance for the transplantation field.<br><br>This work builds on previous studies that used developmentally relevant mitogens to maintain and expand mouse RPCs by using human cells, a photoreceptor reporter line and deeper transcriptome profiling to characterize the cells. However, for the study to represent a significant advance the authors need to do more to describe how this system is an improvement over the previous mouse RPC expansion studies where multipotentiality was not maintained and to provide more evidence of multilineage differentiation.<br><br>1. The authors should do more to describe why their method is an improvement over previous methods to maintain RPC multipotentiality. Many of the "multipotency" genes are not unique to RPCs. Moreover, bulK RNA-seq does not address the extent to which RPCs co-express these determinants.<br>**This point regarding novelty was mentioned by several other reviewers,**

	**and the lack of scRNA-seq data (or rather, shortcomings of bulk RNA-seq) should be outlined as a limitation, for *Communications Biology*.**<br><br>2. The choice of growth factors/mitogens is based on the literature, but how were dosages optimized and what is the evidence that each supplement is required and activates the anticipated signaling pathway. The choice of a GSK3 inhibitor is not well justified, because in some contexts canonical Wnt activation in the mammalian retina is growth inhibitory.<br>**This point was also raised by other reviewers. Please justify the dosage and clarify how these factors were chosen.**<br><br>3. Lineage specification is not deep enough to be convincing. While the evidence that Crx-mCherry and Nrl are co-expressed is suggestive of a rod cell fate these cells should be more fully characterized in terms of morphology, proliferation status, photoreceptor differentiation markers and competence for differentiation. The authors describe the cells as being transplant competent (line 360), but this is not demonstrated here.<br>**These points were also raised by Reviewer #3, and should be addressed for further consideration at *Communications Biology*.**<br><br>4. Other lineages. The conclusion that the culture system maintains multipotentiality should be supported with more data showing specification of other lineages. There is no evidence here for cone, bipolar or MG differentiation.<br>**If feasible, please include evidence for at least one other cell type, or expand on the limitations of this model (also, per points from Reviewer #3).**<br><br>5. If bona fide photoreceptor induction does take place in these cultures it would be very helpful to know how robust this induction is. For example, how many photoreceptors are specified as a function of the starting number of proliferating RPCs. If only a few cells survive after mitogen withdrawal then this will not be a useful resource for photoreceptor production.

Reviewer #2 information

**Expertise**	This reviewer has expertise in retinal organoids.
**Editor's comments**	This reviewer finds the topic to be interesting, but lacking in terms of demonstrated translational relevance, and emphasizes the need for inclusion of other cell lines to improve reproducibility (among other experiments to infer cell identity or maturity).

Reviewer #2 comments

**Section**	**Annotated Reviewer Comments**
**Remarks to the Author: Overall significance**	The goal of this study was to optimize a protocol to obtain neuroretinal progenitors with the capacity to generate the different retinal neurons and bankable them for later use in cell therapies.<br><br>**General comments**<br>The overall concept is interesting because it addresses the problem to obtain, store and use homogeneous retinal cell progenitors in regenerative medicine. The manuscript is well written, and the study contained novel data regarding the gene expression profile of RPC after several passes confirming the expression of multipotent and neurogenic genes. Plagiarism <20%<br><br>The main flaws of this study are several:<br><br>1- The novelty is not high since there are clinical trials using banked RPC already.<br>**This point was raised by other reviewers (though not in the context of clinical trials), and should be acknowledged in a revision.**<br><br>2- The usefulness of these cells *in vivo* has also not been proven.<br>**While *Communications Biology* would not expect any *in vivo* experiments as part of a revision, it would be necessary to qualify any translational relevance of the current study.**<br><br>3- The study is performed using only one hiPSC line (AAVS1::CrxP_H2BmCherry62). Usually, protocols are tested in 2 o 3 different lines to corroborate their reproducibility because there exist differences in the differentiation process among lines.<br>**This point was also raised by Reviewer #3. Please include at least one other cell line, for further consideration at *Communications Biology* (though RNA-seq would not be necessary).**

	4- In the point of view of future cell therapies to treat retinal diseases, the use of the RPC at these stages of immaturity could be detrimental rather than beneficial. Instead, it would be more interesting to develop bankable progenitors of specific retinal lineages to focus the cell therapies on the correct cell type affected.<br>**Again, translational applications could be mentioned as a future direction.**<br><br>5- Another issue is that the need to generate RO for 2 weeks is not clear. Authors used retinal organoids to differentiate RPC into more mature retinal cells but after the dissociation and passaging, retinal markers returned to more progenitors similar to RPC or RO W4. Also, they remove pigmented areas from RO, which could be a valid reason for their use, but then the authors used RPC to differentiate towards retinal pigment epithelium. It is not clear the advantage of the RO stage<br>**This point should be addressed for further consideration at *Communications Biology*.**
**Remarks to the Author: Strength of the claims**	**Methods sections:**<br>- Differentiation of hiRPCs line 119: it is not specified the duration of the differentiation towards retinal differentiation.<br>**For the sake of reproducibility, please elaborate on this (and the other points, outlined below) step in the Methods.**<br><br>- **Line 121**: Explain the reason to dissociate the retinal cells before the immunostaining. During passaging, differentiated cells could lose their cellular morphology and molecular signatures.<br><br>- **Line 136:** deltaCt method is used to give the relative expression, not the fold gene expression that corresponds to delta-deltaCT.<br><br>- **Line 161:** Why do authors use gelatin in the blocking buffer instead of serum from whatever animal the secondary antibody is derived from?<br><br>- **Line 172:** SDs abbreviation<br><br>**Results:**<br>1 - Line 181: Is there any reason to dissociate the retinal organoids at W6, only<br><br>2 weeks after the suspension culture and not later? Please, explain the reasons, if any.<br><br>2 - Explain the differences between W4 RPC before the lifting and W4 RO? It seems that they are at the same week of differentiation, then RPC are at the beginning of week 4 and RO at the end? Please, specify it.

3 - It seems that the RPC after passaging grow in colonies, resembling hiPCS. I wonder whether the single-cell passaging did not yield a homogeneous culture instead of the colony-like culture. Please, explain the reasons, if any. Why authors did not use any marker of pluripotency?

4 - Line 183: “The internal layer contains PAX6+/VSX2- cells, corresponding to RGCs, the first post-mitotic differentiated cell type. The external layer is composed of a cell population co-expressing PAX6+/VSX2+ and the mitotic marker KI67, representing RPCs” From this, some questions arise:

- Did you check if the PAX6+/VSX2- cells corresponded to RGC in your organoids? Maybe using beta-III tubulin?
- If the ROs are dissociated completely, the resulting retinal progenitor cell culture is a mix of retinal progenitors with the ability to differentiate toward photoreceptor, bipolar, Müller, and a high % of RGC with the ability to generate only GC. SO, I would expect to have a portion of the cells that are not CRX-mCherry+ after the differentiation with the specific medium. This is not shown in any figure, but instead, PAX6+ cells differentiated mainly to interneurons (comment 8)

Please include additional staining for these markers, for further consideration at *Communications Biology*.

5 - **Suppl Fig. 1:** At W6, RAX is not detected in the outer layer of the developing organoids by IHC (at this point Rax is usually highly expressed there), but it seems there is some expression on the inner GRC layer. Explain it, please.

6 - **Line 229:** PC2 indicates a reversion in the RPC population closer to W4 RO, my question is why is necessary to develop RO for 2 weeks instead of using RPC at W4? Did the authors study the expression of markers at W4 to see if there is the possibility to subculture RPC without the RO stage? This is further confirmed in line 237: “This result is consistent with those of the PCA, confirming that the expanded RPC population corresponded to retinal progenitors 239 at a similar "progenitor stage" as that found in W4 ROs”. (Again, see comment 2 regarding W4 RPC or W4 RO). And also in line 268.

7- There are no pluripotency markers used to corroborate that hiRPC cells did not regain pluripotency, which is primordial for the safety of their further use in cell transplantation.

Please incorporate these pluripotency markers, for further consideration at *Communications Biology*.

8- **Line 290 and 307:** in line 183 authors specified that PAX6+/VSX2- cells corresponded to RGC but in Figure 3 the double staining showing PAX6+/BRN3a+ cells is only true for a few PAX6+ cells, and conversely, PAX6+ cells are all positive for AP2 or LIM, which suggests that all PAX6+ differentiate mainly to interneurons (shown in fig 3b) and not ganglion cells.

	9- in BM medium, it is only characterized the expression of BRN3A marker. What about the expression of the rest of the retinal cells?<br><br>**Figures:**<br>- Statistics are only included in 2 graphs out of 14.<br>**For the sake of reproducibility, please include more details about statistical comparisons in each figure legend. Please refer to the Open Research Evaluation at the end of this document for more guidance on statistical reporting and general recommendations to improve reproducibility.**<br><br>- **Fig 1B,D and I:** these images are also repeated in the supplementary Figure 1 and 2.<br>**Please do not repeat images between figures.**<br><br>- At W4, the ROs are already formed if it is the time when are lifted? (see comment 2 in results)<br><br>- Suppl. Figure 6 is not in the additional data, guess that if Suppl. Fig 5<br>**Please clarify if this is a typo, or a missing figure.**<br><br>**Discussion:**<br>**Line 358:** “Expanded hiRPCs can be directed to produce photoreceptor precursor-enriched cultures of up to 90% without a purification step” However, the original expanded RPC contained PAX6+ cells that are not differentiated towards photoreceptors. The % of PAX6+ cells in the original hiRPC is not shown. So, 90% is a high percentage of photoreceptors without any purification step.
**Remarks to the Author: Reproducibility**	The statistical analysis is missing in most of the results. Can't be judged. Some images are repeated in supplementary data, maybe other images could be used.

Reviewer #3 information	
**Expertise**	This reviewer has expertise in retinal organoids and development.
**Editor's comments**	This reviewer echoes several concerns regarding the use of a single cell line, and believes the method could be useful if put in proper context of former studies, and incorporates additional validation. They also raise several useful suggestions to help qualify the text.

Reviewer #3 comments

Section	Annotated Reviewer Comments
**Remarks to the Author: Overall significance**	Gozlan et al. present in their manuscript culture methods for expansion of a mixed population of cells primarily containing multipotent retinal progenitor cells from human retinal organoids at early stages of differentiation. Authors characterize these cells using immunocytochemistry and gene expression analyses following isolation, limited expansion in a specific culture medium and banking. Moreover, RPCs within this cell population are capable of generating early photoreceptors and RPE cells, which may be used for cell therapy studies. The quality of data is good and the manuscript overall is well written. Isolation and banking of RPCs from human organoids is an interesting strategy, especially for researchers working on retinal cell therapy, and could be implemented in future studies, but the authors should better discuss prior work along similar lines and limitations of their approach.
**Remarks to the Author: Impact**	Authors present an interesting application of progenitor cells present in retinal organoids towards developing cell therapies and drug screening. The work builds upon and expands from previous studies, but overall novelty is somewhat limited.
**Remarks to the Author: Strength of the claims**	**Major criticisms:**<br>1. It appears that only one cell line has been used to validate methods presented in the study, for this protocol to be useful as a research tool it has to be validated with additional cell lines (at least 1-2).<br>**At least one additional cell line should be included, for further consideration at *Communications Biology*.**<br><br>2. Photoreceptor and RPE differentiation has been performed to early, immature stages. It is not clear from the presented data if mature cell phenotypes such as visual opsin expression in photoreceptors or apico-basal polarity and transepithelial resistance features of RPE cells can be achieved from cells obtained using this method.

Minor comments:
Line 18 - authors should avoid the expression 'for the first time' in this context since some aspects of the study based on prior work and not entirely novel, for instance cryopreservation of whole human organoids has been described;

In general, we recommend avoiding terms like “new” or “novel”, as novelty should be made clearly purely from context.

Line 44 - other methods have been described such as for generation of neurospheres previously, authors may describe it as a novel method for specific expansion of RPCs;

Line 61 - method should be verified using at least one if not two additional hESC or iPSCs lines;

Line 124 – typo, should be PFA;

Line 159 – again, should be PFA;

Lines 182-184 - post-mitotic RGCs are not a subpopulation of progenitors, authors need to clarify that not performing a progenitor purification step results in incorporating early-postmitotic RGCs in the resulting final population;

Lines 194-197 - What's proportion of proliferating cells within this population? Cell cycle analysis using flow cytometry would be ideal or immunostaining for proliferation marker such as Ki67 or PCNA followed by quantification;

This point was also raised by Reviewer #1; please be sure to report proliferation rates and/or additional immunostaining.

Line 262 - Authors should comment that this could be a result of changing from free floating to adherent culture conditions;

Line 265 - Are W6 organoids cultured in RPCM showing signatures of reset to an early multipotent RPC state?;

Lines 268-269 - Authors should comment that dissociation, freezing and replating could trigger this reset to more proliferative state, post-mitotic cells could be more fragile and selectively lost during the procedures;

Lines 293-302 - Do these photoreceptor precursor mature expressing opsins and other phototransduction components with further culture?

Lines 322-323 - Functional assays for RPE tight junctions and apico basal polarity such as transepithelial resistance measurement or culture on transwells and detection of selective secretion of factors such PEDF would provide further evidence for differentiation into mature RPE phenotype;

This point would not be necessary for further consideration at *Communications Biology*, though could be mentioned as a limitation or future direction.

Line 323 - CRX is known to be expressed in human RPE, so perhaps surprising that reporter expression was not observed;

Line 326 - mixed populations including RPCs since there is no purification step;

Line 328 - Authors only perform short term differentiation experiments, not clear if cells generated using this method can achieve any significant levels of maturation such as expression of visual opsins by photoreceptors;

Line 335 - Authors also demonstrate maintenance of RPC multipotency in a very limited number of passages (up to 4);

Please acknowledge this point as a limitation of the study.

Line 339 - Effect of individual factors and their synergistic/additive effects are not really presented or discussed;

This point was raised by several other reviewers.

Line 354 - Differentiation into RGCs and interneurons from banked cells has not been conclusively demonstrated, since RGCs were lost past 1 week differentiation in BM medium and results on interneurons such as amacrine cell are mostly absent. Authors should comment on limitations of their method in producing RGCs;

This point would be necessary for further consideration at *Communications Biology*.

Line 357 - Method should be validated with additional 1-2 iPSC/hESC lines, not clear how reproducible is this protocol;

Lines 361-364 - Data presented suggests that this method is not suitable for generation of RGCs;

Lines 368-370 - Are the numbers after gene names refer to citations? Then they need to be put in superscript

Line 372 - This statement is misleading, authors use an established human pluripotent stem cell line to differentiate retinal progenitors, the cell line has been previously published, it is not new;

Figure 1 - Proportions of cells expressing RPC and proliferation markers should be quantified;

Figure 4 - Data on apico-basal polarity and transepithelial resistance would

	provide further evidence of utility of this method to generate functional, mature RPE;<br><br>**Supplemental Fig. 3 -** How was proliferation measured? There is no description of method used. How many biological replicates were used? Are the results statistically significant?<br><br>**Supplemental Fig. 4 -** Again, number of biological replicates and statistical significance of results is missing;
**Remarks to the Author: Reproducibility**	Authors appear to use on a single stem cell line, which is not sufficient to validate a method. Biological replicates are often not clearly defined, number of independent cultures/ samples used needs to clarified.

<table>
<tr><th colspan="2">Reviewer #4 information</th></tr>
<tr><td>Expertise</td><td>This reviewer has expertise in retinal development, degeneration, and repair.</td></tr>
<tr><td>Editor's comments</td><td>While the reviewer finds the study to be of great interest, they also share concerns regarding the limited rationale for the differentiation protocol that should be addressed in a revision.</td></tr>
<tr><th colspan="2">Reviewer #4 comments</th></tr>
<tr><th>Section</th><th>Annotated Reviewer Comments</th></tr>
<tr><td>Remarks to the Author: Overall significance</td><td>Gozlan et al. report methods and media for the generation, expansion, and cryopreservation of human induced pluripotent stem-cell derived-RPCs (hiRPCs). They provide evidence that thawed and passed hiRPCs maintained biochemical and transcriptional RPC phenotypes and their ability to differentiate into photoreceptor precursor, ganglion, horizontal, amacrine or retinal pigmented epithelial cells. They also performed RNA-seq analysis and identified transcriptomic similarities and differences between hiRPCs and retinal organoids. Overall, the manuscript is well written, reports original findings, and will be of interest to others in the field.

Major points
One major finding in the manuscript is the identification of five extrinsic factors that maintain the cell state of retinal progenitor cells in cultures. However, they do not describe clearly how they end up with these five factors at such concentrations although they provide references of these five factors in the discussion. Have the authors assessed the effect of each factor on the phenotype of retinal progenitor cells? Have they removed one factor, such as CHIR99021, and then examined the outcome?
<blockquote>While it wouldn't be necessary to show what happens when one factor is removed, generally more rationale/justification for this differentiation protocol should be included for further consideration at Communications Biology.</blockquote>The culture grown under the aforementioned condition appears to be a mixed population, which is evidenced by the expression of VSX2 and PAX6 (Figure 1i). Therefore, the culture is not clonal. In addition, have they examined the phenotype in higher passages? Can it be called a cell line?

Photoreceptor precursor cells that are differentiated from thawed cells are still at immature stages. Can these precursor cells differentiate into photoreceptors with inner and outer segments? Without these mature structures, these</td></tr>
</table>

	photoreceptor precursor cells may not have sufficient predictive power in testing.<br><br>**Minor points**<br>The fonts in several figures are too small in the printout. Please increase the font size in Figs. 1, 2.
**Remarks to the Author: Impact**	The manuscript is suitable for *Communications Biology* after major revision.
**Remarks to the Author: Strength of the claims**	These two points need to be addressed:<br><br>Have the authors assessed the effect of each factor on the phenotype of retinal progenitor cells? Have they removed one factor, such as CHIR99021, and then examined the outcome?<br><br>Photoreceptor precursor cells that are differentiated from thawed cells are still at immature stages. Can these precursor cells differentiate into photoreceptors with inner and outer segments?<br>**This point could be addressed as part of the rebuttal to point #4 from Reviewer #1**
**Remarks to the Author: Reproducibility**	Statistical analysis and the quality of the data are reasonably good.

Open research evaluation

Guidelines for Transparency and Openness Promotion (TOP) in Journal Policies and Practices ("TOP Guidelines")

The recommendations and requests in the table below are aimed at bringing your manuscript in line with common community standards as exemplified by the TOP Guidelines. While every publisher and journal will implement these guidelines differently, the recommendations below are all consistent with the policies at Nature Portfolio. In most cases, these will align with TOP Guidelines Level 2.

FAIR Principles

The goal of the recommendations in the table below related to **data or code** availability is to promote the FAIR Guiding Principles for scientific data management and stewardship (*Scientific Data* **3**: 160018, 2016). The FAIR Principles are a set of guidelines for improving 4 important aspects of digital research objects: **F**indability, **A**ccessibility, **I**nteroperability and **R**eusability.

ORCID

ORCID is a non-profit organization that provides researchers with a unique digital identifier. These identifiers can be used by editors, funding agencies, publishers, and institutions to reliably identify individuals in the same way that ISBNs and DOIs identify books and articles. Thus the risk of confusing your identity with another researcher with the same name is eliminated. **The ORCID website** provides researchers with a page where your comprehensive research activity can be stored.

Springer Nature collaborates with the ORCID organization to ensure that your research contributions (as authors and peer reviewers) are correctly attributed to you. Learn more at https://www.springernature.com/gp/researchers/orcid

Data availability
**Data Availability statement**
Many journals, including all Nature Portfolio journals, require a Data Availability Statement in the manuscript as a condition of publication. The Data Availability Statement should be as detailed as possible and include accession codes or other unique IDs for deposited data, information about where source data can be found, and specify any restrictions to data access that may apply. At a minimum, the statement should indicate that data are available upon request and explain how data access can be granted. If data access is not possible, the reasons for this must be made clear in the Data Availability Statement.
Thank you for including a Data Availability statement. While you have included some important information, the editors have noted that some details appear to be missing.The Data Availability Statement should be as detailed as possible and include accession codes or other unique IDs for deposited data, information about where source data can be found, and specify any restrictions to data access that may apply. At a minimum, the statement should indicate that data are available upon request and explain how data access can be granted. If data access is not possible, the reasons for this must be made clear in the Data Availability statement.<br>● More information about the Nature Portfolio data availability policy can be found here<br>● More information about formatting Data Availability Statements can be found here
**Mandatory data deposition**
Most scientific journals, including all Nature Portfolio journals, require that any newly-generated RNA sequence data must be made publicly available before publication. There are some exceptions allowed for sensitive clinical data, but this should be discussed with the editor. All data must be deposited in a community-approved repository and accession codes/unique IDs must be included within the Data Availability statement in the manuscript.<br><br>Examples of appropriate public repositories are listed below:<br>● Gene Expression Omnibus (Microarray or RNA sequencing data)<br>● Sequence Read Archive (high-throughput sequence data)<br>● The European Nucleotide Archive (ENA)<br>More information on mandatory data deposition policies at the Nature Portfolio can be found at http://www.nature.com/authors/policies/availability.html#data<br><br>Please visit this link for a list of approved repositories for various data types.

Other data requests
We strongly encourage the deposition of your full microscopy image data sets in the Image Data Resource: https://idr.openmicroscopy.org/about
All source data underlying the graphs and charts presented in the main figures must be made available as Supplementary Data (in Excel or text format) or via a generalist repository (eg, Figshare or Dryad). This is mandatory for publication in a Nature Portfolio journal, but is also best practice for publication in any venue.<br><br>**The following figures require associated source data:** Fig 1c, 1e, 1g-h, 2e-f, 3c-d, 3f-h, 3j, 4d
**Data citation**
Please cite (within the main reference list) any datasets stored in external repositories that are mentioned within their manuscript. For previously published datasets, we ask that you cite both the related research article(s) and the datasets themselves. For more information on how to cite datasets in submitted manuscripts, please see our data availability statements and data citations policy:<br>https://www.nature.com/documents/nr-data-availability-statements-data-citations.pdf<br><br>Citing and referencing data in publications supports reproducible research, by increasing the transparency and provenance tracking of data generated or analysed during research. Citing data formally in reference lists also helps facilitate the tracking of data reuse and may help assign credit for individuals' contributions to research. A number of Springer Nature imprints are signatories of the Joint Declaration on Data Citation Principles, which stress the importance of data resources in scientific communication.
**Ethics**
Thank you for providing a Competing Interests statement. It appears that not all authors are mentioned; please ensure your Competing Interests statement includes information about all authors.<br><br>See the Nature Portfolio competing interests policy for further information:<br>https://www.nature.com/nature-research/editorial-policies/competing-interests<br>The Springer Nature policy can be found here:<br>https://www.springernature.com/gp/policies/editorial-policies<br><br>**Please relabel "Disclosure of potential conflicts of interest" as "Competing interests", and be sure to list which authors do (or do not) have any competing interests.**

We encourage authors to follow the principles laid out in the 2016 ISSCR Guidelines for Stem Cell Research and Clinical Applications of Stem Cells (https://www.isscr.org/policy/guidelines-for-stem-cell-research-and-clinical-translation).

To adhere to community standards, and as a prerequisite for publication in a Nature Portfolio journal, studies that report experiments involving the use of human embryos and gametes, human embryonic stem cells and related materials, and clinical applications of stem cells must include confirmation that all experiments were performed in accordance with relevant guidelines and regulations.

Please ensure your manuscript includes an ethics statement identifying the institutional and/or licensing committees approving the experiments and describing any relevant details. The ethics statement must also confirm that informed consent was obtained from all recipients and/or donors of cells or tissues, where necessary, and describe the conditions of donation of materials for research, such as human embryos or gametes.

Further details on our policies can be found at https://www.nature.com/commsbio/editorial-policies/ethics-and-biosecurity

Reporting & reproducibility

Cell line misidentification and cross-contamination is a common problem with serious consequences. Authors are asked to report on the source and authentication of their cell lines.

We believe that research publications should adhere to high standards of transparency and robustness in their methods and results. This, in turn, supports the principle of reproducibility, which is a foundation of good research, especially in the natural sciences.

The Methods section should contain sufficient detail such that the work could be repeated. It is preferable that all key methods be included in the main manuscript, rather than in the Supplementary Information. Please avoid use of "as described previously" or similar, and instead detail the specific methods used, with appropriate attribution.

Please note that Nature Portfolio journals allow unlimited space for Methods.

Materials availability

Nature Portfolio supports the Resource Identification Initiative (https://www.force11.org/group/resource-identification-initiative), with the aim of promoting unique, persistent identification and tracking of key biological resources, including antibodies, cell lines, model organisms and tools.

We encourage authors to include unique identifiers provided by the Resource Identification Portal, (RRIDs; for example, Antibody: RRID:AB_2140114; Organism: RRID:MGI_MGI:3840442), in the manuscript. More information on how to include listed RRIDs or generate new RRIDs can be found on the Resource Identification Portal: https://scicrunch.org/resources/about/Getting%20Started

We strongly encourage deposition of any new cell lines in repositories that will distribute them with certificates of authentication. Alternatively, we recommend that authors establish a profile of their new cell lines to allow future authentication. The distribution of human cell lines used in research should not be hindered by restrictions from donors. Researchers developing cell lines must investigate and disclose any restrictions associated with the tissue they are using.

Statistical reporting

Wherever statistics have been derived (e.g. error bars, box plots, statistical significance) figure legends should provide and define the n number (i.e. the sample size used to derive statistics) as a precise value (not a range), using the wording “n=X biologically independent samples/animals/cells/independent experiments/n= X cells examined over Y independent experiments” etc. as applicable. The figure legends must also indicate the statistical test used. Where appropriate, please indicate in the figure legends whether the statistical tests were one-sided or two-sided and whether adjustments were made for multiple comparisons. For null hypothesis testing, please indicate the test statistic (e.g. F, t, r) with confidence intervals, effect sizes, degrees of freedom and P values noted.

All error bars need to be defined in the figure legends (e.g. SD, SEM) together with a measure of centre (e.g. mean, median). For example, the legends should state something along the lines of “Data are presented as mean values +/- SEM” as appropriate. All box plots need to be defined in the legends in terms of minima, maxima, centre, bounds of box and whiskers and percentile.

When describing results as "significant" in the main text, please include details about the statistical test used and provide an exact p-value, rather than a significance threshold.

Please note that statistics such as error bars significance and p values cannot be derived from n<3 and must be removed in all such cases.

We strongly discourage deriving statistics from technical replicates, unless there is a clear scientific justification for why providing this information is important. Conflating technical and biological variability, e.g., by pooling technically replicates samples across independent

experiments is strongly discouraged.

For examples of expected description of statistics in figure legends, please see the following:
https://www.nature.com/articles/s41467-019-11636-5 or
https://www.nature.com/articles/s41467-019-11510-4

To improve reproducibility of your analyses, please provide details regarding:

- Treatment of outliers
- Methods used for data fitting and provide a rationale for this approach

Data presentation

Bar graphs should only be used to present counts or proportions. If you are using bar graphs that present means/averages, it is best practice **to include individual data points** and/or convert the graph to a boxplot or dot-plot. You may wish to refer to this blog post (https://ecrlife420999811.wordpress.com/2018/07/10/beyond-bar-graphs-free-tools-and-resources-for-creating-more-transparent-figures-for-small-datasets/) about representing data distribution in plots (particularly for small datasets).

Please update Fig 1c, 1e, 1g-h, 3c-d, 3f-h, 3j, 4d, as well as Supp Fig 3b, 3d-e, 4a-b accordingly.

Please ensure that all microscopy images and photographs include a scale bar and this scale bar is defined on the panels or in the figure legends.
