# Supplementary material for: Bankable human iPSC-derived retinal progenitors represent a valuable source of multipotent cells
Source: Commun Biol. 2023 Jul 21;6:762. doi: 10.1038/s42003-023-04956-2 (PMC10362027; doi:10.1038/s42003-023-04956-2)
Supplement: Supplementary file 2 — Supplementary Information [file 42003_2023_4956_MOESM2_ESM.pdf]

Supplementary Information

Supplementary Figure 1: Identification of retinal ganglion cells in W6 ROs.

Supplementary Figure 2: Characterization of RPC population in W4, W5, and W6 ROs.

Supplementary Figure 3: Analysis of the individual molecule activities present in the RPC-dedicated medium and characterization of hiRPCp0 generated from hiPSC-2 and hiPSC-5F lines.

Supplementary Figure 4: Immunofluorescence staining of pluripotency markers for the hiRPCs.

Supplementary Figure 5: Characterization of thawed hiRPCs at different passages.

Supplementary Figure 6: Characterization of the hiRPCs generated from the hiPSC-2 line.

Supplementary Figure 7: Multipotency and proliferation analysis of hiRPCp2 cultured in RPCM or BM.

Supplementary Figure 8: RNAseq gene validation by RT-qPCR and Venn Diagram of DEGs of hiRPCp2 vs W4 ROs vs W6 ROs.

Supplementary Figure 9: Spontaneous differentiation of hiRPCp2-4 in BM.

Supplementary Figure 10: Differentiation of hiRPCs generated from hiPSC-2 line.

Supplementary Figure 11: Differentiation of hiRPCs into late retinal cell types.

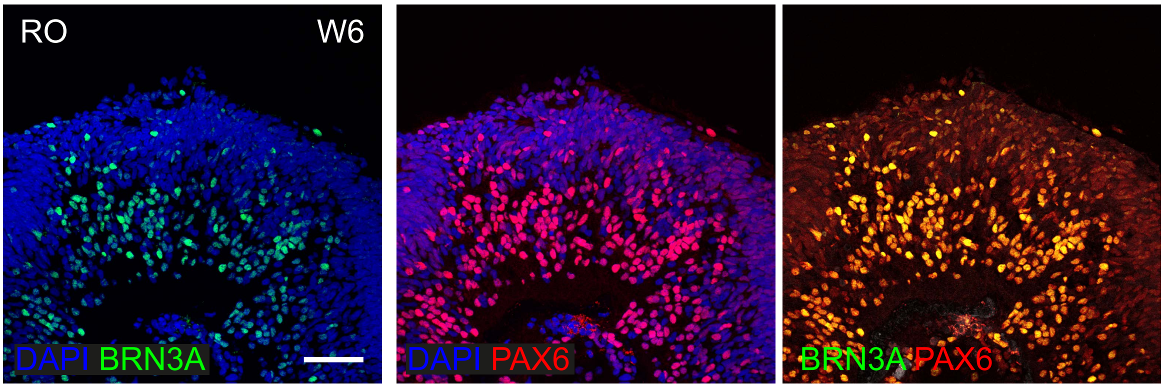

Supplementary Figure 1: Identification of retinal ganglion cells in W6 ROs. Immunofluorescence staining for BRN3A and PAX6, and mCherry endogenous staining of cryosectioned W6 ROs. Nuclei were counterstained with DAPI (blue). hiPSC-5FC-derived RO. Scale bar: 50 µm.

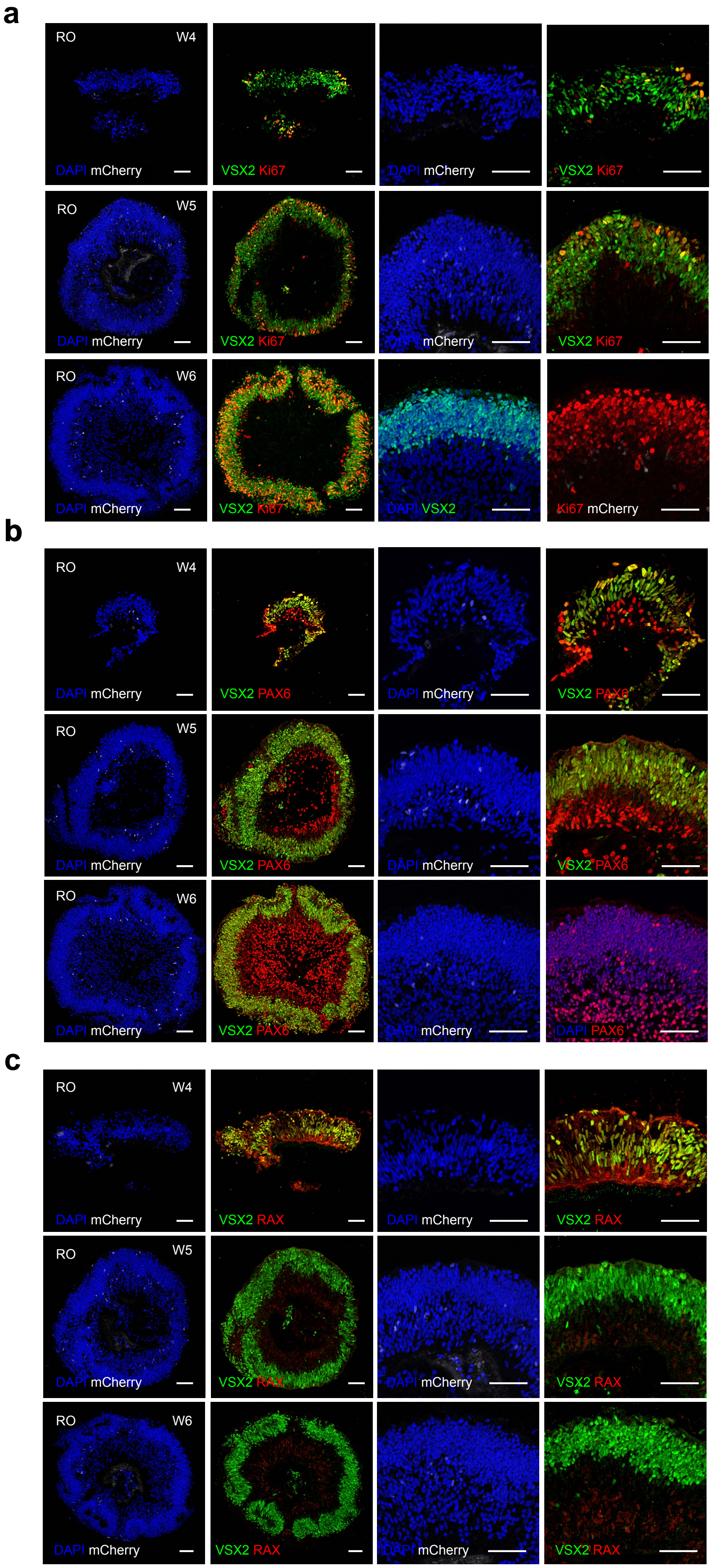
a
RO
W4
DAPI mCherry
VSX2 Ki67
DAPI mCherry
VSX2 Ki67
RO
W5
DAPI mCherry
VSX2 Ki67
DAPI mCherry
VSX2 Ki67
RO
W6
DAPI mCherry
VSX2 Ki67
DAPI VSX2
Ki67 mCherry
b
RO
W4
DAPI mCherry
VSX2 PAX6
DAPI mCherry
VSX2 PAX6
RO
W5
DAPI mCherry
VSX2 PAX6
DAPI mCherry
VSX2 PAX6
RO
W6
DAPI mCherry
VSX2 PAX6
DAPI mCherry
DAPI PAX6
c
RO
W4
DAPI mCherry
VSX2 RAX
DAPI mCherry
VSX2 RAX
RO
W5
DAPI mCherry
VSX2 RAX
DAPI mCherry
VSX2 RAX
RO
W6
DAPI mCherry
VSX2 RAX
DAPI mCherry
VSX2 RAX

Supplementary Figure 2: Characterization of RPC population in W4, W5, and W6 ROs. **A,** Immunofluorescence staining for VSX2 and Ki67, and mCherry endogenous staining of cryosectioned W4, W5, and W6 ROs. **b,** Immunofluorescence staining for VSX2 and PAX6, and mCherry endogenous fluorescence of cryosectioned W4, W5, and W6 ROs. **c,** Immunofluorescence staining for VSX2 and RAX, and mCherry endogenous staining of cryosectioned W4, W5, and W6 ROs. Nuclei were counterstained with DAPI (blue). Stars indicate images used in Fig. 1. hiPSC-5FC-derived ROs. Scale bar: **a, b, c,** 50 µm.

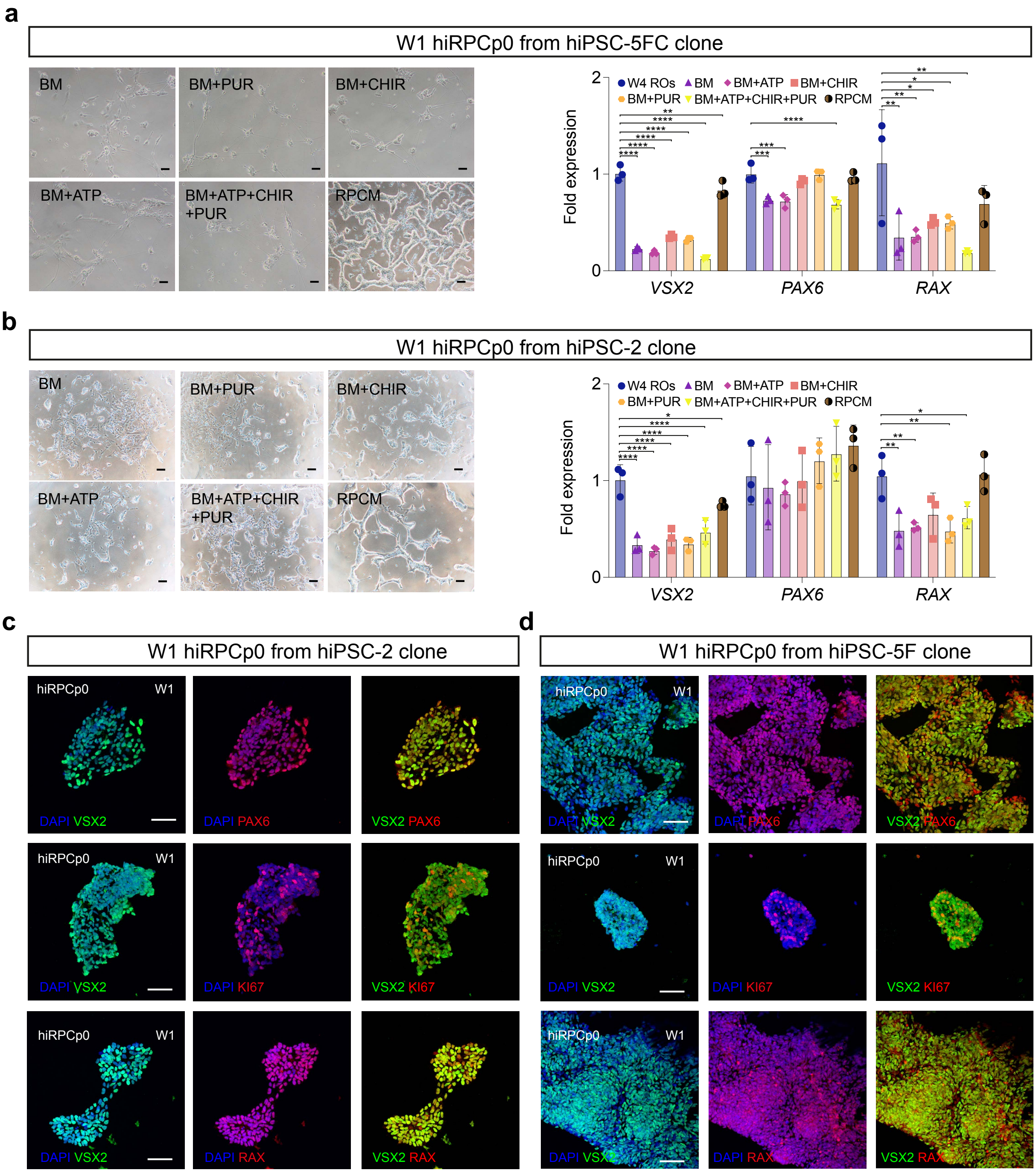

Supplementary Figure 3: Analysis of the individual molecule activities present in the RPC-dedicated medium and characterization of hiRPCp0 generated from hiPSC-2 and hiPSC-5F lines. a, Phase-contrast and brightfield microscopy of hiRPCs cultured in basal medium (BM), BM+PURMORPHAMINE (BM+PUR), BM+CHIR99021 (BM+CHIR), BM+ATP, BM+ATP+PUR+CHIR and RPCM and RT-qPCR analysis of *VSX2*, *PAX6* and *RAX* in hiRPCp0 generated from the hiPSC-5FC line. Data are normalized to that of W4 ROs and presented as mean ± SD (n = 3 per time point). **b,** Phase-contrast and brightfield

microscopy of hiRPCs cultured in basal medium (BM), BM+PURMORPHAMINE (BM+PUR), BM+CHIR99021 (BM+CHIR), BM+ATP, BM+ATP+PUR+CHIR and RPCM and RT-qPCR analysis of VSX2**,** PAX6 and RAX in hiRPCp0 generated from the hiPSC-2 line. Data are normalized to that of W4 ROs and presented as mean ± SD (n = 3 per time point). **c,** Immunofluorescence staining for VSX2, PAX6 and RAX of W1 hiRPCp0 generated from the hiPSC-2 line. **d,** Immunofluorescence staining for VSX2, PAX6 and RAX of W1 hiRPCp0 generated from the hiPSC-5F line. One-way ANOVA followed by a Dunnett's multiple comparison test. Comparison to W4 ROs. ****p<0,0001; ***p < 0.001; **p < 0.01; *p < 0.05. Nuclei were counterstained with DAPI (blue). **a,** hiPSC-5FC-derived cells; **b, c,** hiPSC-2-derived cells; **d,** hiPSC-5F-derived cells. Scale bar: a, b, **c**, **d**, 50 µm.

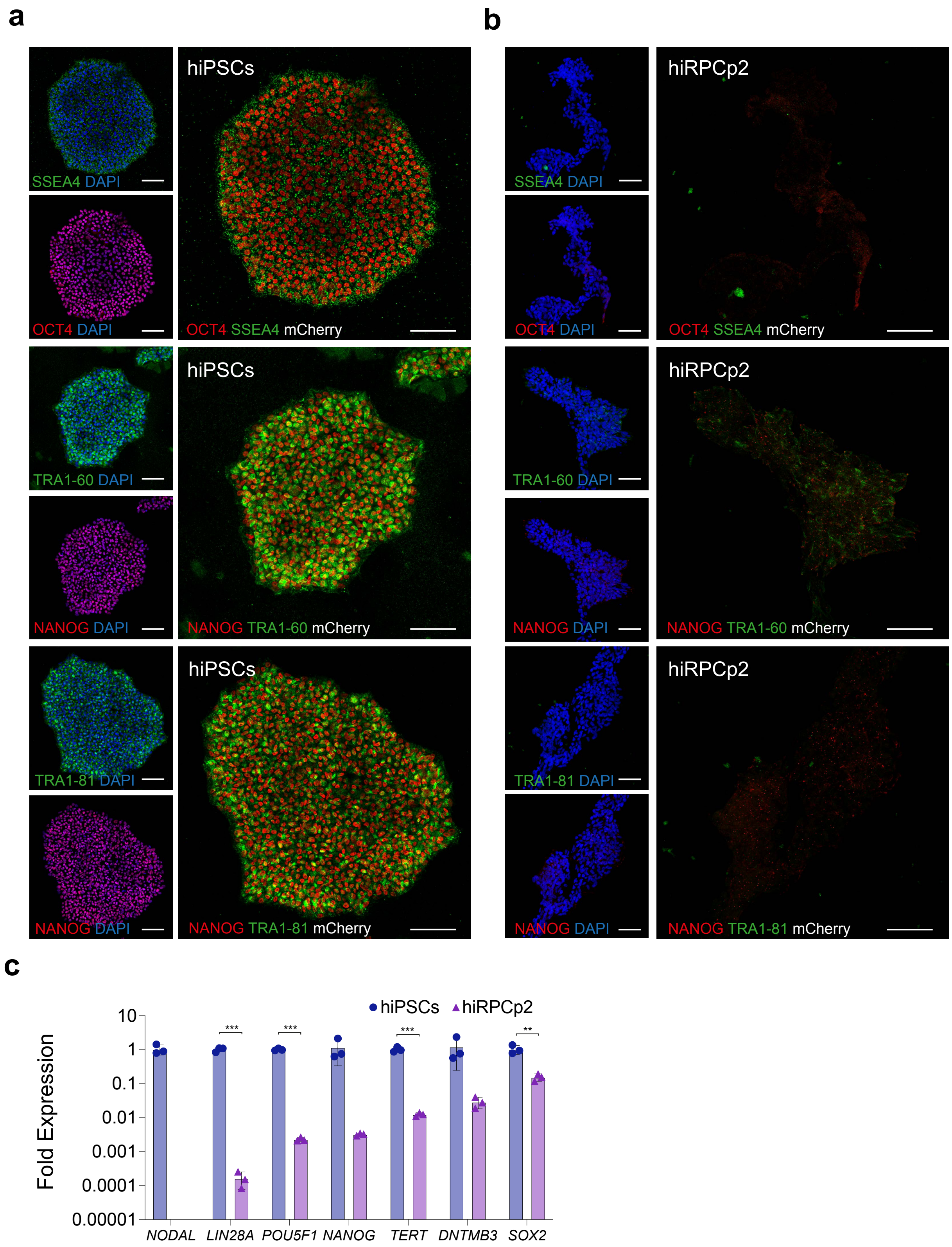

Supplementary Figure 4: Immunofluorescence staining of pluripotency markers for the hiRPCs. a, Immunofluorescence staining of pluripotency markers (SSEA4, OCT4, NANOG, TRA1-60 and TRA1-81) for the hiPSC-5FC. **b,** Immunofluorescence staining of pluripotency markers (SSEA4, OCT4, NANOG,

TRA1-60 and TRA1-81) for the hiRPCp2 generated from the hiPSC-5FC line. **c,** RT-qPCR analysis of pluripotency and self-renewal markers in hiPSC-5FC and derived hiRPCp2. Data are normalized to that of hiPSCs and presented as mean ± SD (n = 3 per time point). Two-tailed Student's t-test. ***p < 0.001; **p < 0.01. Nuclei were counterstained with DAPI (blue). hiPSC-5FC-derived cells. Scale bar: **a,** 100 μM; **b,** 50 μM.

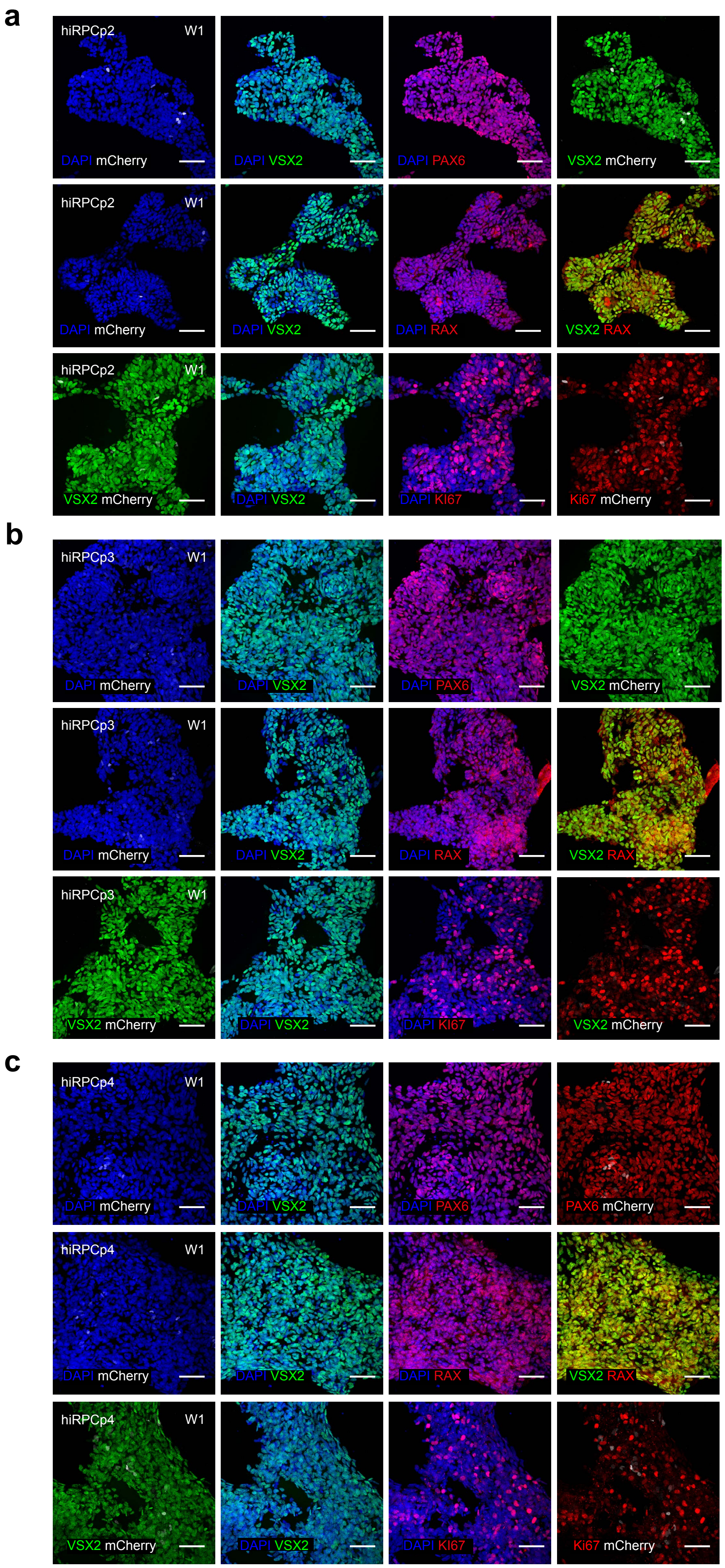
a
hiRPCp2
W1
DAPI mCherry
DAPI VSX2
DAPI PAX6
VSX2 mCherry
DAPI RAX
VSX2 RAX
DAPI Ki67
Ki67 mCherry
b
hiRPCp3
c
hiRPCp4
PAX6 mCherry

Supplementary Figure 5: Characterization of thawed hiRPCs at different passages. a, Endogenous mCherry and immunofluorescence staining of RPCM-cultured hiRPCp2 at W1 post passage for VSX2, PAX6, RAX, and Ki67. **b,** Endogenous mCherry and immunofluorescence staining of RPCM-cultured hiRPCp3 at W1 post passage for VSX2, PAX6, RAX, and Ki67. **c,** Endogenous expression of mCherry and immunofluorescence staining of RPCM-cultured hiRPCp4 at W1 post passage for VSX2, PAX6, RAX, and Ki67. Nuclei were counterstained with DAPI (blue). hiPSC-5FC-derived cells. Scale bar: **a**, **b**, **c**, 50 μm.

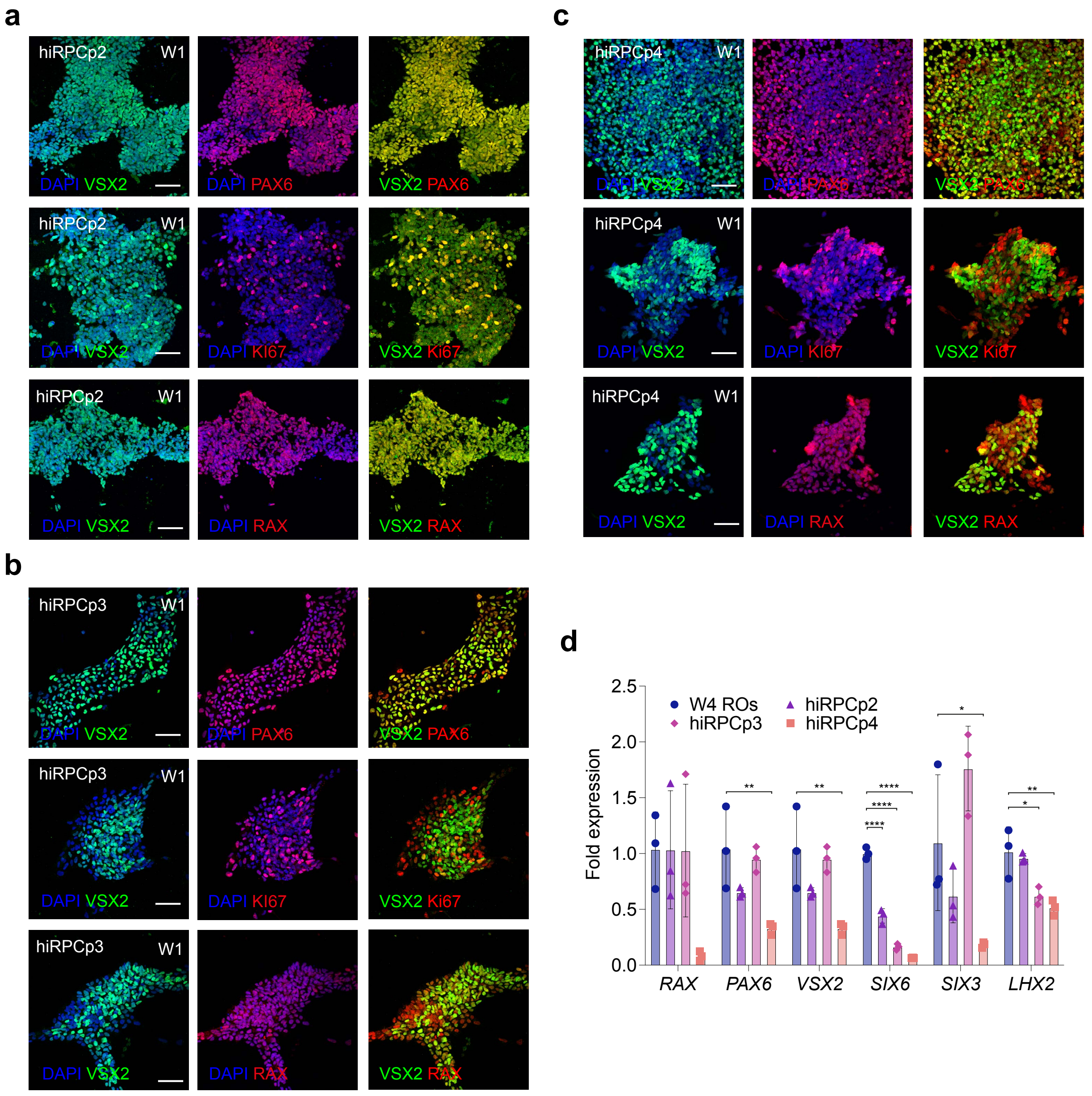

Supplementary Figure 6: Characterization of the hiRPCs generated from the hiPSC-2 line. a, Immunofluorescence staining of thawed hiRPCp2 at W1 VSX2, PAX6, RAX, and Ki67. **b,** immunofluorescence staining of hiRPCp3 at W1 post passage for VSX2, PAX6, RAX, and Ki67. **c,** Immunofluorescence staining of hiRPCp4 at W1 post passage for VSX2, PAX6, RAX, and Ki67. **d,** RT-qPCR analysis of EFTFs (*RAX, PAX6, SIX6, SIX3, LHX2*) and *VSX2* in hiRPCp2 to hiRPCp4. Data are normalized to that of W4 ROs and presented as mean ± SD (n = 3 per time point). One-way ANOVA followed by a Dunnett's multiple comparison test. Comparison to W4 ROs. ****p<0,0001; **p < 0.01;

*p < 0.05. Nuclei were counterstained with DAPI (blue). hiPSC-2-derived cells. Scale bar: **a**, **b**, **c**, 50 µm.

Stat.

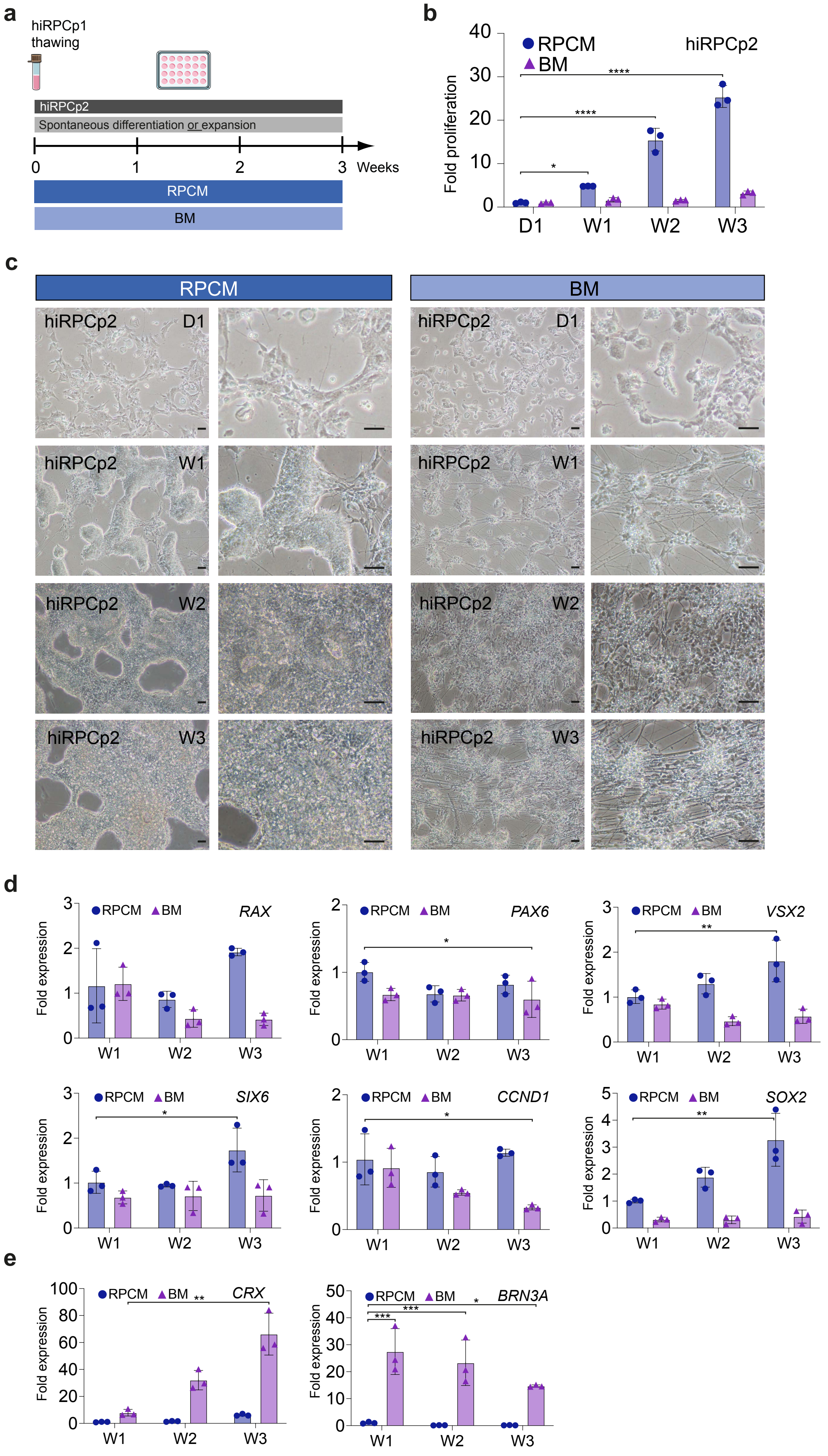
a
hiRPCp1 thawing
hiRPCp2
Spontaneous differentiation or expansion
0
1
2
3
Weeks
RPCM
BM
b
RPCM
BM
hiRPCp2
Fold proliferation
D1
W1
W2
W3
c
RPCM
BM
hiRPCp2 D1
hiRPCp2 W1
hiRPCp2 W2
hiRPCp2 W3
d
RAX
PAX6
VSX2
SIX6
CCND1
SOX2
Fold expression
e
CRX
BRN3A
Fold expression

Supplementary Figure 7: Multipotency and proliferation analysis of hiRPCp2 cultured in RPCM or BM. a, Schematic diagram illustrating hiRPCp2 expansion in RPCM or BM for three weeks without passage from cryopreserved hRPCp1. **b,** Proliferation analysis of hiRPCp2 cultured in RPCM or BM after W1, W2, and W3. Data are normalized to that of D1 and presented as mean ± SD (n = 3 per time point). **c,** Phase-contrast images of hiRPCp2 cultured in RPCM or BM at day 1 (D1), W1, W2, and W3. **d,** RT-qPCR analysis of EFTFs (*RAX, PAX6, VSX2, SIX6*) , *SOX2*, and *CCDN1* in hiRPCp2 cultured in RPCM or BM at W1 , W2, and W3. **e,** RT-qPCR analysis of specific photoreceptor (*CRX)* and RGC (*BRN3A)* markers. All data were normalized to expression of the gene in hiRPCp2 cultured in RPCM at W1. Data are normalized to that of W1 RPCM and presented as mean ± SD (n = 3 per time point). One-way ANOVA followed by a Dunnett's multiple comparison test. Comparison to D1 (b) or W1 (d,e). ***p < 0.001; **p < 0.01; *p < 0.05. hiPSC-5FC-derived cells. Scale bar: **c,** 100 µM.

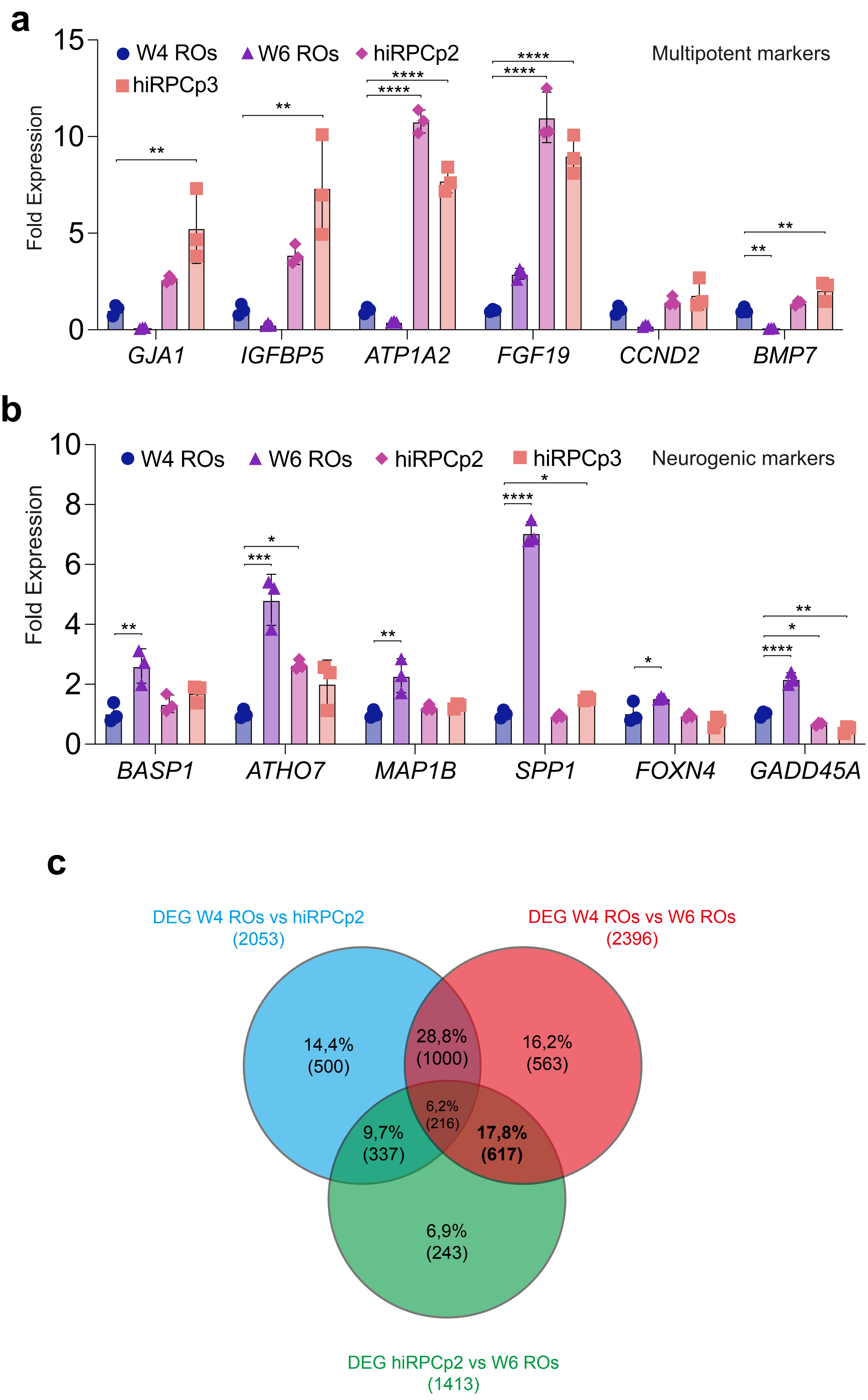
a
Fold Expression
W4 ROs
W6 ROs
hiRPCp2
hiRPCp3
Multipotent markers
GJA1
IGFBP5
ATP1A2
FGF19
CCND2
BMP7
b
Fold Expression
W4 ROs
W6 ROs
hiRPCp2
hiRPCp3
Neurogenic markers
BASP1
ATHO7
MAP1B
SPP1
FOXN4
GADD45A
c
DEG W4 ROs vs hiRPCp2
(2053)
DEG W4 ROs vs W6 ROs
(2396)
14,4%
(500)
28,8%
(1000)
16,2%
(563)
6,2%
(216)
9,7%
(337)
17,8%
(617)
6,9%
(243)
DEG hiRPCp2 vs W6 ROs
(1413)

Supplementary Figure 8: RNAseq gene validation by RT-qPCR and Venn Diagram of DEGs of hiRPCp2 vs W4 ROs vs W6 ROs. a, RT-qPCR analysis of the multigenic gene markers *GJA1, IGFBP5, ATP1A2, FGF19, CCND2*, and *BMP7*. Data are normalized to that of W4 ROs and presented as mean ± SD (n = 3 per time point). **b,** RT-qPCR analysis of the neurogenic gene markers *BASP1, ATOH7, MAP1B, SPP1, FOXN4*, and *GADD45A*. Data are normalized to that of W4 ROs and presented as mean ± SD (n = 3 per time point). All data were normalized to the expression of the gene in W4 ROs, composed mainly of RPCs. **c,** This representation highlights 617 genes varying in expression between W4 and W6 ROs and between hiRPCp2 and W6 ROs, but not between W4 ROs and hiRPCp2. One-way ANOVA followed by a Dunnett's multiple comparison test. Comparison to W4 ROs. ****$p < 0.0001$, ***$p < 0.001$; **$p < 0.01$; *$p < 0.05$. hiPSC-5FC-derived cells.

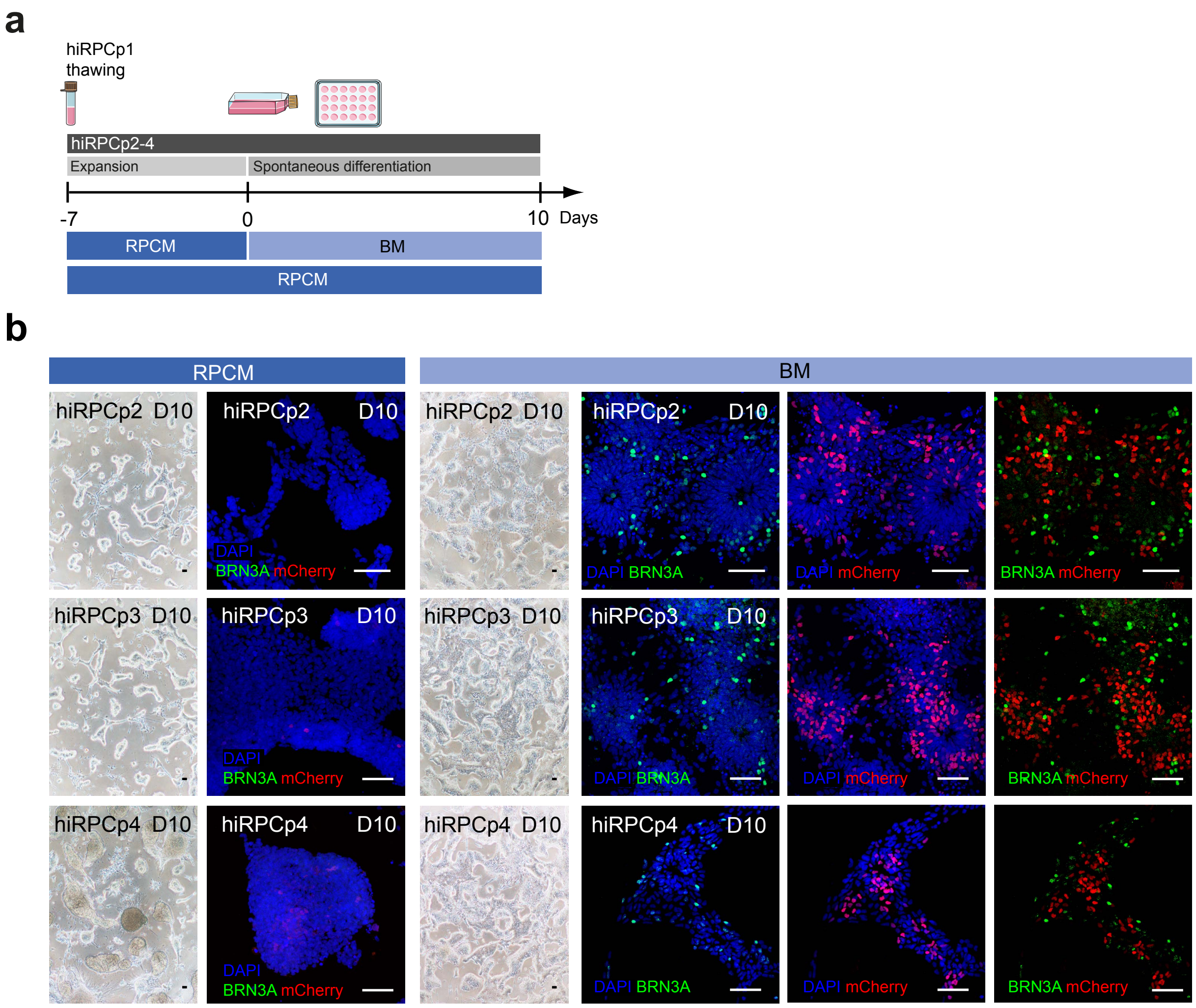

Supplementary Figure 9: Spontaneous differentiation of hiRPCp2-4 in BM. a, Schematic diagram illustrating hiRPCp2-4 from cryopreserved hRPCp1 cultured in RPCM or BM. **b,** Phase-contrast image and immunofluorescence staining for BRN3A (RGCs) and endogenous expression of mCherry in hiRPCp2 to hiRPCp4 after 10 days (D10) in BM or RPCM. Nuclei were counterstained with DAPI (blue). hiPSC-5FC-derived cells. Scale bar: **b,** 50 µM.

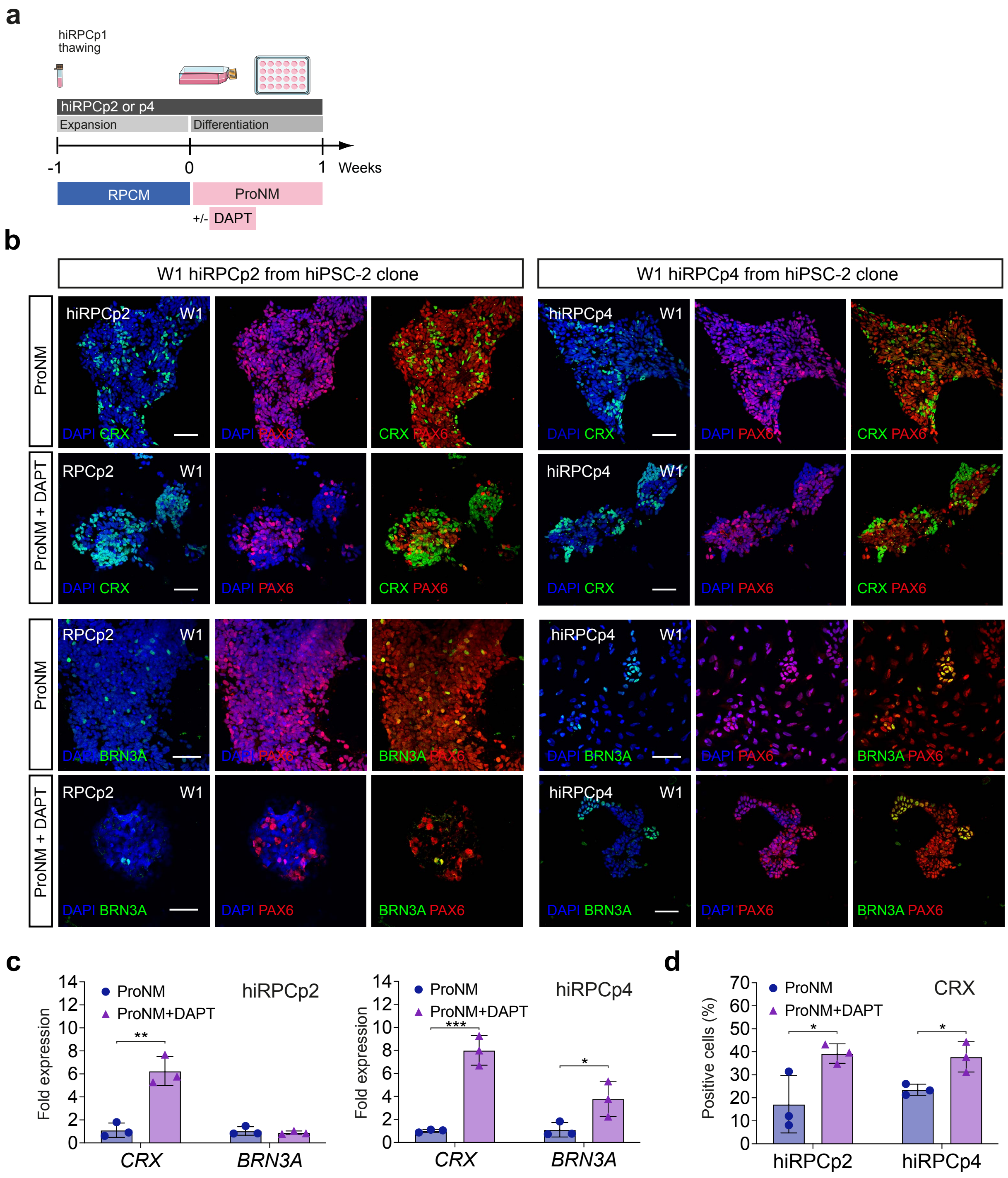

Supplementary Figure 10: Differentiation of hiRPCs generated from hiPSC-2 line. a, Schematic diagram illustrating the differentiation protocol to generate early retinal cell types. **b,** Immunofluorescence staining of differentiated hiRPCp2 and hiRPCp4 after one week (W1) of culture in ProNM ± DAPT for CRX (PPCs), BRN3A (RGCs), and PAX6. **c,** RT-qPCR analysis of *CRX* and *BRN3A* in hiRPCp2 and hiRPCp4 differentiated in ProNM ± DAPT at W1. Data were normalized to that of differentiated hiRPCp2 or hiRPCp4 at W1 and presented as mean ± SD (n = 3 per time point). d, High-

content analysis of CRX^+ cells in adherent cultures of hiRPCp2 and hiRPCp4 differentiated in ProNM ± DAPT at W1. hiPSC-2-derived hiRPCs. Two-tailed Student's t-test. ***$p < 0.001$; **$p < 0.01$; *$p < 0.05$. Nuclei were counterstained with DAPI (blue). hiPSC-2-derived cells. Scale bar: **b,** 50 µM.

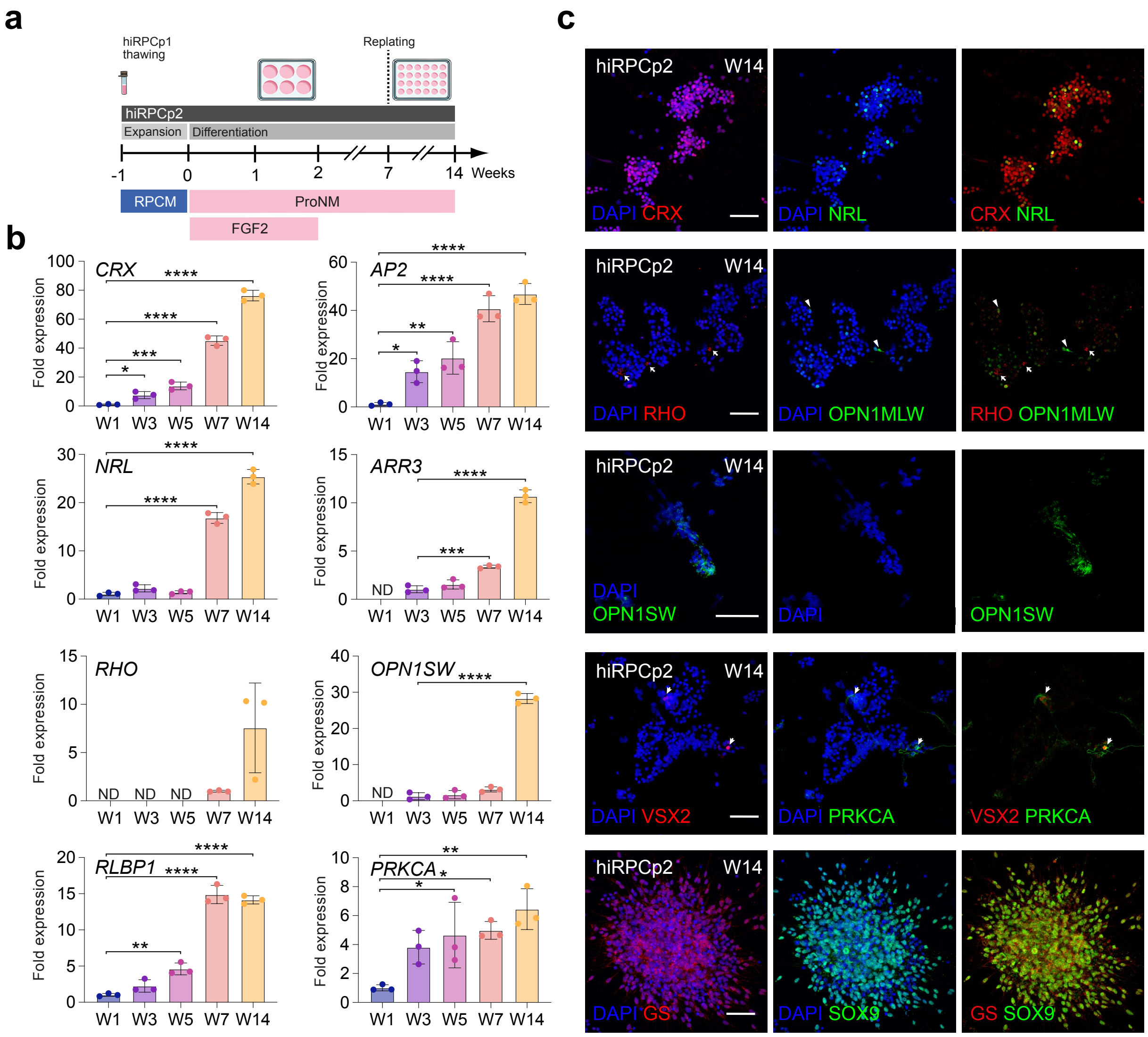

Supplementary Figure 11: Differentiation of hiRPCs into late retinal cell types. a, Schematic diagram illustrating the differentiation protocol to generate late retinal cell types in adherent culture condition. **b,** RT-qPCR analysis of CRX (PPCs), AP2 (amacrine cells), NRL (rod PPCs), ARR3 (cone PPCs), RHODOPSIN (RHO, rod photoreceptors), BLUE OPSIN (OPN1SW, blue cone photoreceptors), RLBP1 (Müller glial cells), PRKCA (bipolar cells) in hiRPCp2 at W1, W3, W5, W7 and W14. Data are normalized to that of W1 (*CRX, NRL, AP2, RLBP1, PRKCA*), W3 (*ARR3, OPN1SW*) or W7 (*RHO*) and presented as mean ± SD (n = 3 per time point). c, Immunofluorescence staining of differentiated hiRPCp2 at W14 for CRX, NRL, RHO, RED/GREEN OPSIN (OPN1MLW, red/green cone photoreceptors), OPN1SW, VSX2, PRKCA (bipolar cells) and GLUTAMINE SYNTHETASE (GS), SOX9 (Müller glial cells). One-way ANOVA followed by a Dunnett's multiple comparison test (b; *CRX, NRL, AP2, RLBP1, PRKCA, ARR3, OPN1SW*) or two-

tailed Student's t-test (b; *RHO*). Comparison to W1 (*CRX, NRL, AP2, RLBP1, PRKCA*), W3 (*ARR3, OPN1SW*) or W7 (*RHO*). ****p<0,0001; ***p < 0.001; **p < 0.01; *p < 0.05. Nuclei were counterstained with DAPI (blue). hiPSC-5F-derived cells. Scale bar: **b,** 50 µM.
