# Supplementary material for: Bankable human iPSC-derived retinal progenitors represent a valuable source of multipotent cells
Source: Commun Biol. 2023 Jul 21;6:762. doi: 10.1038/s42003-023-04956-2 (PMC10362027; doi:10.1038/s42003-023-04956-2)
Supplement: Supplementary file 3 — Supplementary Data 1 [file 42003_2023_4956_MOESM3_ESM.docx]

| **Supplementary Data 1 : List of Taqman Gene Expression ID Assays used for qRT-PCR** | |
| --- | --- |
| **Gene Symbols** | **Assays IDs (Life technology)** |
| *18S* | 18S-Hs99999901_s1 |
| *AP2* | TFAPA Hs01029413_m1 |
| *ARR3* | ARR3-Hs00182888_m1 |
| *ATOH7* | ATOH7-Hs00376955_s1 |
| *BASP1* | BASP1-Hs00932356_s1 |
| *BEST1* | BEST1-Hs00188249_m1 |
| *BLUE OPS* | OPN1SW-Hs00181790_m1 |
| *BMP7* | BMP7-Hs00233476_m1 |
| *BRN3A* | POU4F1-Hs00366711_m1 |
| *BRN3B* | POU4F2-Hs00231820_m1 |
| *CCND1* | CCND1-Hs00765553_m1 |
| *CCND2* | CCND2 Hs00153380_m1 |
| *CRX* | CRX-Hs00230899_m1 |
| *DNTMB3* | DNMT3B-Hs00171876_m1 |
| *FGF19* | FGF19 Hs00192780_m1 |
| *FOXN4* | FOXN4-Hs01566111_m1 |
| *GADD45A* | GADD45A-Hs00169255_m1 |
| *GJA1* | GJA1- Hs00748445_s1 |
| *IGFBP5* | IGFBP5 Hs01052295_m1 |
| *LHX2* | LHX2-Hs00180351_m1 |
| *LHX1* | LHX1-Hs00232144_m1 |
| *LIN28A* | LIN28A-Hs00702808_s1 |
| *MAP1B* | MAP1B-Hs01067016_m1 |
| *MERTK* | MERTK-Hs01031973_m1 |
| *MITF* | MITF-Hs01117294_m1 |
| *NANOG* | NANOG-Hs02387400_g1 |
| *NODAL* | NODAL-Hs00415443_m1 |
| *NRL* | NRL-Hs00172997_m1 |
| *PAX6* | PAX6-Hs00240871_m1 |
| *PEDF* | SERPINF1-Hs01106934_m1 |
| *POU5F1(OCT-4)* | POU5F1-Hs00999632_g1 |
| *PRKCA* | POU5F1-Hs00999632_g1 |
| *RAX* | RAX-Hs00429459_m1 |
| *RLBP1* | POU5F1-Hs00999632_g1 |
| *RHO* | RHO-Hs00892431_m1 |
| *SIX3* | SIX3-Hs00193667_m1 |
| *SIX6* | SIX6 Hs00201310_m1 |
| *SOX2* | SOX2-Hs01053049_s1 |
| *SSP1* | SSP1- Hs00959010 |
| *TERT* | TERT-Hs00972656_m1 |
| *VSX2* | VSX2-Hs00766959_s1 |
