# Supplementary material for: Bankable human iPSC-derived retinal progenitors represent a valuable source of multipotent cells
Source: Commun Biol. 2023 Jul 21;6:762. doi: 10.1038/s42003-023-04956-2 (PMC10362027; doi:10.1038/s42003-023-04956-2)
Supplement: Supplementary file 4 — Supplementary Data 2 [file 42003_2023_4956_MOESM4_ESM.docx]

**Supplementary Data 2 : List of antibodies used for immunohistochemistry analysis.**

|  |  |  |  |
| --- | --- | --- | --- |
| **Antigen** | **Species** | **Dilution** | **Source** |
| *Primary antibodies* | | | |
| AP2 | Mouse monoclonal | 1/100 | DSHB (3B5) |
| BLUE OPSIN | Rabbit polyclonal | 1/500 | Millipore (AB5407) |
| BRN3A | Mouse monoclonal | 1/250 | Millipore (MAB1585) |
| CRX | Mouse monoclonal | 1/5000 | Abnova (H00001406-M02) |
| GLUTAMIN SYNTHASE | Mouse monoclonal | 1/500 | Millipore (MAB 302) |
| Ki67 | Rabbit polyclonal | 1/200 | Millipore (AB15580) |
| LHX2 | Goat polyclonal | 1/100 | Santa Cruz (SC19344) |
| LIM1 (LHX1) | Mouse monoclonal | 1/20 | DSHB(4F2-s) |
| MITF | Mouse monoclonal  (clone C5/D5) | 1/400 | Sigma Aldrich (284M-94) |
| NANOG | Rabbit polyclonal | 1/2000 | Cell Signaling (D73G4) |
| NRL | Goat polyclonal | 1/400 | BioTechne (AF2945) |
| OCT4 | Rabbit Polyclonal | 1/100 | Cell Signaling (C30A3) |
| PAX6 | Rabbit polyclonal | 1/2000 | Millipore (AB2237) |
| PAX6 | Mouse monoclonal | 1/1000 | DSHB (AB_528427) |
| PRKCA | Rabbit Polyclonal | 1/1000 | Rabbit Polyclonal |
| RAX/RX | Rabbit polyclonal | 1/50 | Abcam (AB23340) |
| RED/GREEN OPSIN | Rabbit polyclonal | 1/500 | Millipore (AB5405) |
| RHDDOPSIN | Mouse monoclonal | 1/250 | Millipore (MABN15) |
| SSEA4 | Mouse monoclonal | 1/200 | Cell Signaling (MC813) |
| SOX9 | Rabbit polyclonal | 1/1000 | Millipore (AB5535-100UG) |
| TRA1-60 | Mouse monoclonal | 1/100 | Cell Signaling (9656) |
| TRA1-81 | Mouse mononclonal | 1/100 | Cell signaling (9656) |
| VSX2 | Mouse monoclonal | 1/200 | Santa Cruz (SC365519) |
| VSX2/CHX10 | Goat polyclonal | 1/2000 | Santa Cruz (SC1960) |
| ZO-1 | Rabbit polyclonal | 1/250 | Life technologies ; Invitrogen  (61-73000) |
| *Secondary antibodies* | | | |
| Alexa fluor 488 anti-goat | Donkey | 1/300 | Jackson ImmunoResearch  715-545-147 |
| Alexa fluor 488 anti-mouse | Donkey | 1/300 | Jackson ImmunoResearch  715-545-150 |
| Alexa fluor 488 anti-rabbit | Donkey | 1/300 | Jackson ImmunoResearch  711-545-152 |
| Alexa fluor 647 anti-mouse | Donkey | 1/300 | Jackson ImmunoResearch  715-605-150 |
| Alexa fluor 647 anti-rabbit | Donkey | 1/300 | Jackson ImmunoResearch  711-605-152 |
